# One-step generation of composite soybean plants with transgenic roots by *Agrobacterium rhizogenes*-mediated transformation

**DOI:** 10.1186/s12870-020-02421-4

**Published:** 2020-05-12

**Authors:** Ying-lun Fan, Xing-hui Zhang, Li-jing Zhong, Xiu-yuan Wang, Liang-shen Jin, Shan-hua Lyu

**Affiliations:** College of Agriculture, Liaocheng University, Liaocheng, 252000 China

**Keywords:** Hairy root transformation, *Agrobacterium rhizogenes*, Soybean (*Glycine max* (L.) Merr.), Composite plants, *Rfg1*, Strain K599, *YAO* promoter, Tomato (*Solanum lycopersicum*), *Sinorhizobium fredii* USDA193

## Abstract

**Background:**

*Agrobacterium rhizogenes*-mediated (ARM) transformation is a highly efficient technique for generating composite plants composed of transgenic roots and wild-type shoot, providing a powerful tool for studying root biology. The ARM transformation has been established in many plant species, including soybean. However, traditional transformation of soybean, transformation efficiency is low. Additionally, the hairy roots were induced in a medium, and then the generated composite plants were transplanted into another medium for growth. This two-step operation is not only time-consuming, but aggravates contamination risk in the study of plant-microbe interactions.

**Results:**

Here, we report a one-step ARM transformation method with higher transformation efficiency for generating composite soybean plants. Both the induction of hairy roots and continuous growth of the composite plants were conducted in a single growth medium. The primary root of a 7-day-old seedling was decapitated with a slanted cut, the residual hypocotyl (maintained 0.7-1 cm apical portion) was inoculated with *A*. *rhizogenes* harboring the gene construct of interest. Subsequently, the infected seedling was planted into a pot with wet sterile vermiculite. Almost 100% of the infected seedlings could produce transgenic positive roots 16 days post-inoculation in 7 tested genotypes. Importantly, the transgenic hairy roots in each composite plant are about three times more than those of the traditional ARM transformation, indicating that the one-step method is simpler in operation and higher efficiency in transformation. The reliability of the one-step method was verified by CRISPR/Cas9 system to knockout the soybean *Rfg1*, which restricts nodulation in Williams 82 (Nod-) by *Sinorhizobium fredii* USDA193. Furthermore, we applied this method to analyze the function of *Arabidopsis YAO* promoter in soybean. The activity of *YAO* promoter was detected in whole roots and stronger in the root tips. We also extended the protocol to tomato.

**Conclusions:**

We established a one-step ARM transformation method, which is more convenient in operation and higher efficiency (almost 100%) in transformation for generating composite soybean plants. This method has been validated in promoter functional analysis and rhizobia-legume interactions. We anticipate a broad application of this method to analyze root-related events in tomato and other plant species besides soybean.

## Background

Soybean (*Glycine max* (L.) Merr.) is one of the most important crops worldwide accounting for the high content of edible oil (18–20%) and high protein (35–40%) in its seeds. At present, over one-third of edible oils and two-thirds of protein meal are derived from soybean in the world [1, 2]. In 2017, more than 340 million metric tons of soybean were produced globally and provided for ever-increasing human consumption and animal feed necessitates [3]. In the past decades, intensive studies on genetics, multiple omics, and genome-wide association (GWAS) in soybean have been conducted [2, 4–9]. However, a simple and high efficient transformation method is the prerequisite for functional annotations of a large number of candidate genes.

*Agrobacterium rhizogenes*-mediated (ARM) hairy root transformation offers a powerfully transient tool for investigating root biology and has been widely established in a variety of plant species, including the stably genetic transformation-recalcitrant plant species, such as soybean [10, 11]. However, based on the protocols of traditional ARM transformation, stabbing the hypocotyl (widely applied in soybean) and Ex-vitro (or In-vitro), the transformation efficiency is usually low. Furthermore, the transformation method needs two-step operations for generating composite plants, because the induction of hairy roots and the continuous growth of the composite plants were conducted in different media [11–19]. Here, we report a one-step ARM transformation method with higher transformation efficiency for generating composite soybean plants. The induction of hairy roots and the growth of composite plant were finished in a single growth medium. This is a promising technical breakthrough in soybean ARM transformation with simpler operation, higher transformation efficiency, and lower contamination risk in the study on soybean-microbe interactions. In addition, the reliability of the one-step ARM transformation method was validated by CRISPR/Cas9-mediated knockout the resistance to nodulation gene *Rfg1* in the soybean genotype Williams 82 (Nod-) inoculated with *Sinorhizobium fredii* USDA193 [20, 21]. We also applied the one-step transformation method to analyze the expression pattern of the *Arabidopsis YAO* promoter [22] in soybean roots. Moreover, composite tomato plants have been generated by using the one-step ARM transformation method. We anticipate that the one-step transformation method will be widely applied to analyze root-related events in tomato and other plant species besides soybean.

## Results

### Establishment of the one-step ARM transformation method for generating composite soybean plants

**Fig. 1:**
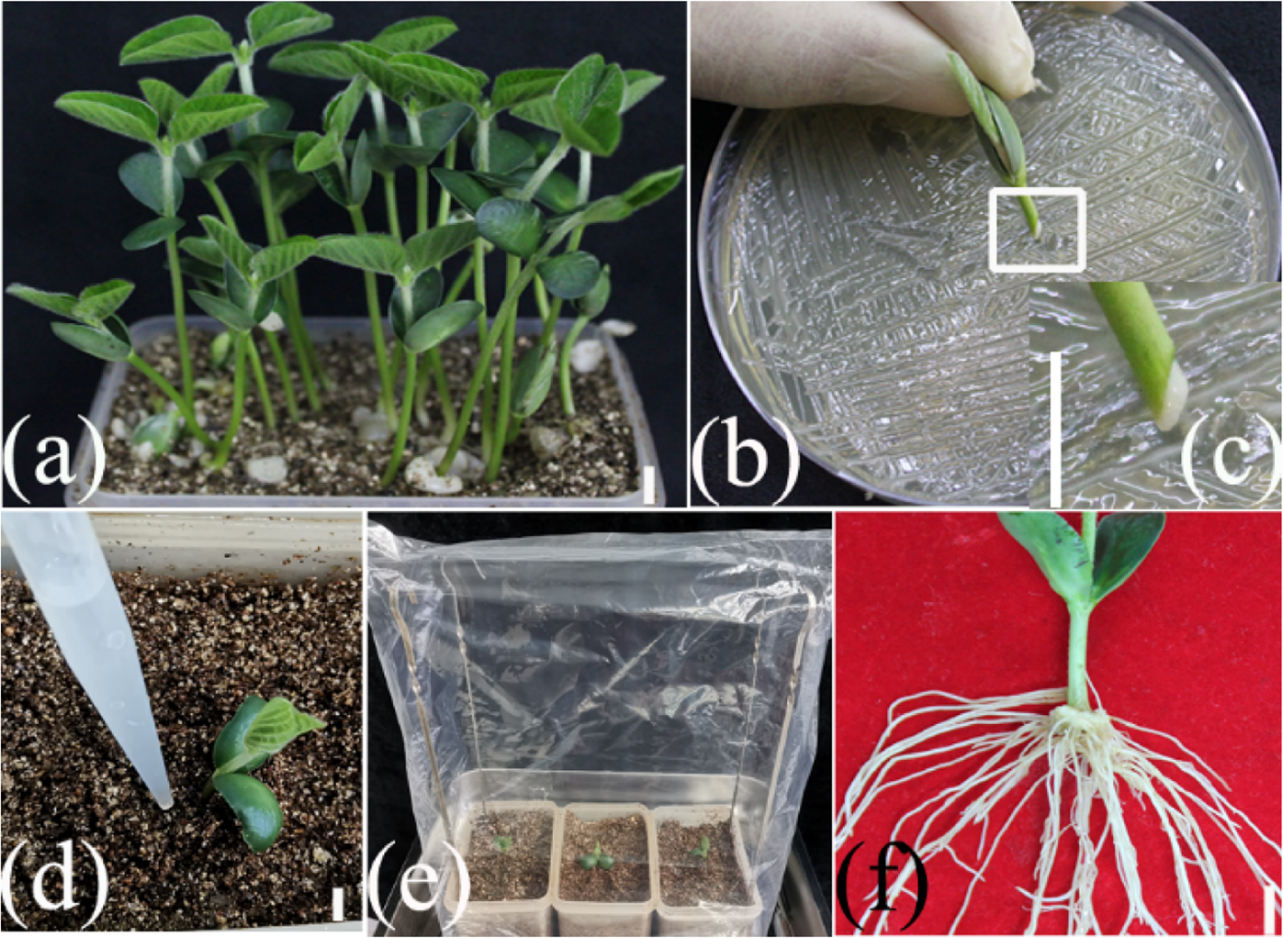
One-step ARM transformation of soybean. 7-day-old seedlings, ideal stage for transformation (**a**); The primary root was cut and the slant cut of hypocotyl (coated the bacterial mass) was scraped on the plate grown K599 (HGCI) (**b**); Close-up of section b marked in the box (**c**); Inoculated explants were planted into vermiculite and watered K599 (HGCI) (**d**); Inoculated seedlings were covered with a plastic bag (**e**); Induced hairy roots at 16 dpi (**f**). Scale bars = 1 cm

**Fig. 2:**
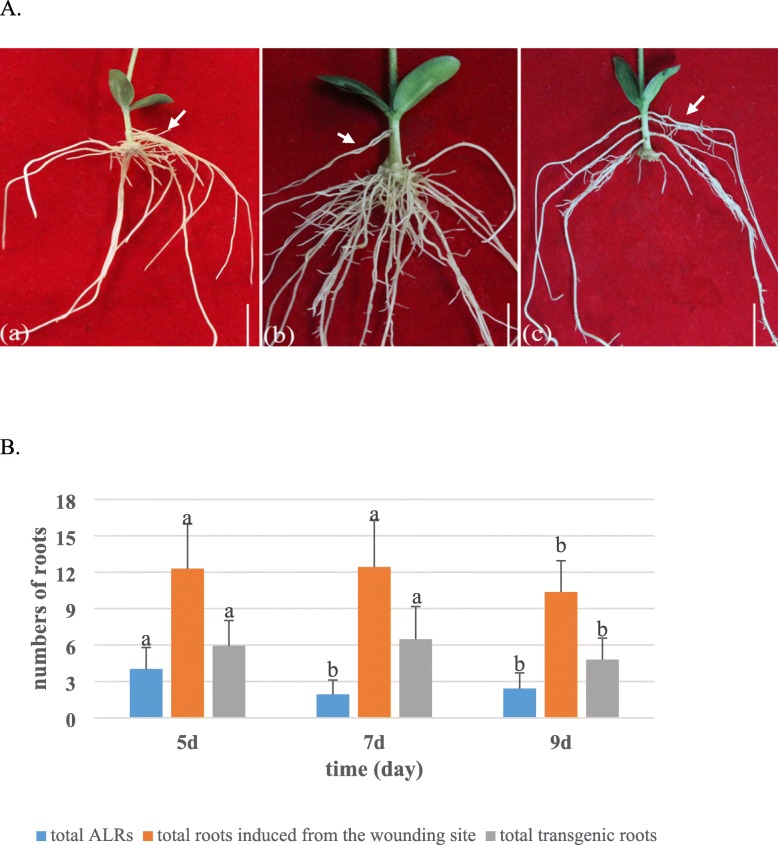
The effects of seedling age on transformation efficiency at 20 dpi. **A**: Hairy roots from 5-day-old (**a**), 7-day-old (**b**) and 9-day-old (**c**) old seedlings, respectively. Arrow indicates ALRs. Bars = 1 cm. **B**: Comparison of total numbers of ALRs, total roots and transgenic roots from different age seedlings

Soybean genotype Williams 82, whose genome has been sequenced completely, was used in this study. The *A*. *rhizogenes* strain K599 was used because the hairy roots can be effectively induced in soybean [11, 23]. A one-step ARM transformation method was developed by cutting the hypocotyl of soybean seedling for generating composite soybean plants. The primary root of a healthy seedling (Fig. 1a) was removed prior to inoculation with the *A*. *rhizogenes* K599 strain harboring the gene construct of interest (K599-HGCI). The slant cut of the residual hypocotyl of seedling was inoculated and coated with the K599-HGCI (Fig. 1b-c). The inoculated seedlings were then directly planted into wet sterile vermiculite and watered with a suspension of K599-HGCI (OD600 = 0.6–1.0, Fig. 1d). Subsequently, the inoculated seedlings were covered with a plastic bag to maintain high humidity in 16 days for hairy roots development (Fig. 1e). Figure 1f indicates the induced hairy roots. In order to optimize the transformation protocol, we tested the effects of seedling age on transformation efficiency. The number of induced hairy roots and transgenic roots in each composite plant were measured for 5-day-, 7-day- and 9-day-old healthy seedlings. The results showed that the 7-day-old seedlings were the best for induction of hairy roots (Fig. 2). As for 5-day-old seedlings, more untransformed adventitious laterals roots (ALRs) and a little lower transformation efficiency were observed compared with the 7-day-old seedlings (Fig. 2a, B). In contrast, both the total numbers of hairy roots and the transgene-positive roots were the lowest for the 9-day-old seedlings (Fig. 2c, B). Therefore, 7-day-old seedlings were used for inoculation in subsequent experiments.

**Fig. 3:**
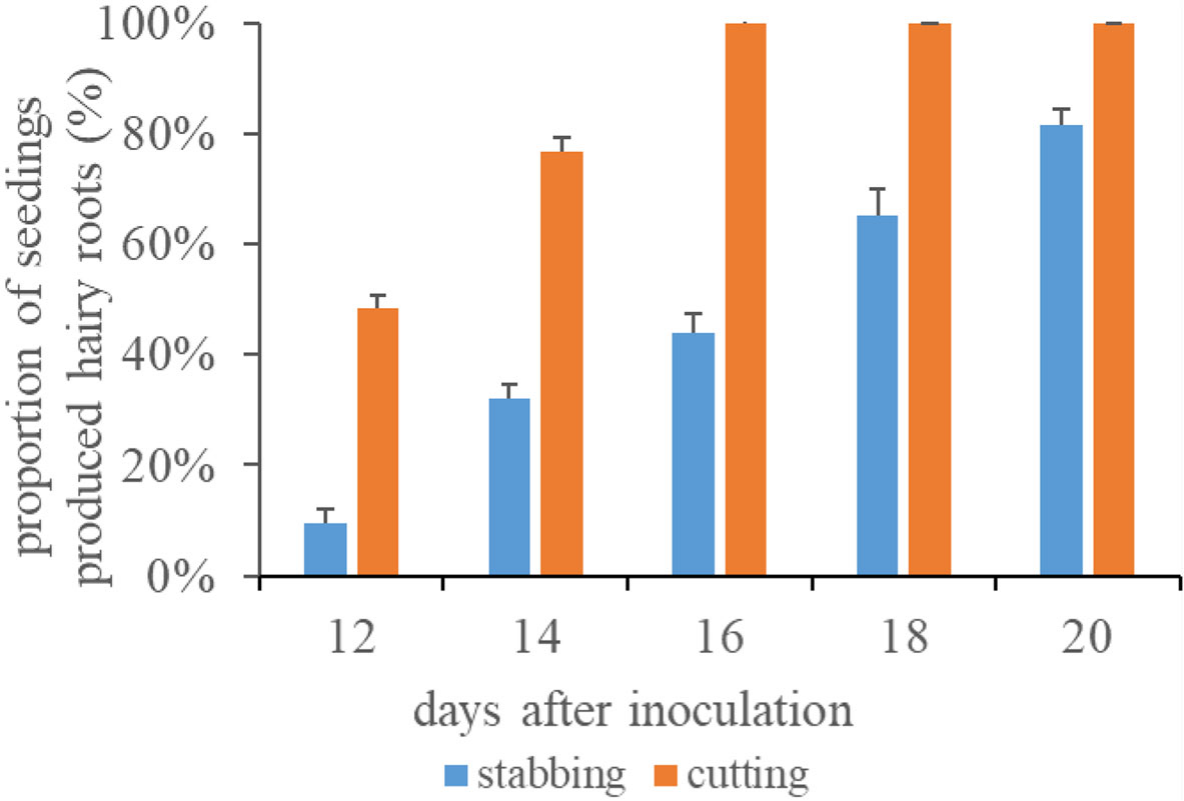
Efficiency of hairy roots induction by different methods. Y-axis represents total numbers of the seedling with at least one hairy root/ total numbers of infected seedlings

Previously, two-step generation of composite soybean plants by ARM transformation by stabbing the hypocotyl of seedlings was widely used [11]. We compared the transformation efficiency of our one-step method with that of two-step method. After 16 days post-inoculation (dpi), 100% of the tested 30 seedlings produced hairy roots when the one-step method was used (Fig. 3). In contrast to the traditional two-step method by stabbing the hypocotyl [11], only 47% of seedlings produced hairy roots at 16 dpi and those reached 80% at 20 dpi (Fig. 3). In both one-step and two-step methods, the total number of induced hairy roots reached the highest value at 20 dpi (data not shown).

**Fig. 4:**
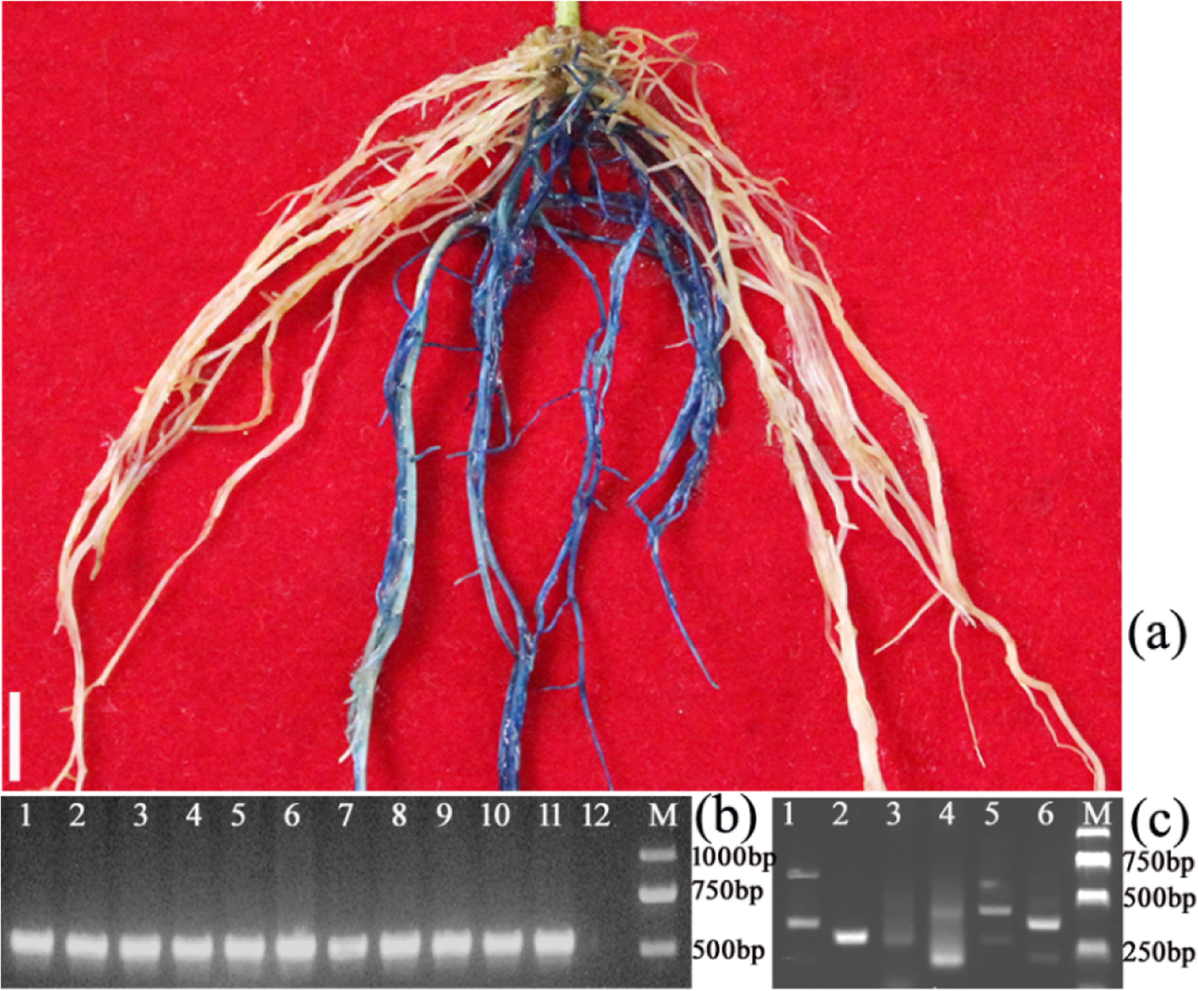
GUS staining and PCR screening of transgenic and non-transgenic hairy roots. **a** GUS staining assay from a composite plant. **b** Validation of GUS-positive roots using PCR. Lanes 1–10, GUS-positive root; Lane 11, K599–1305. Lane 12, GUS-negative root. **c** Amplification of T-DNA flanking sequences by TAIL-PCR. The secondary (Lane 1, 3 and 5) and tertiary TAIL-PCR product (Lane 2, 4 and 6) are shown from independent transgenic roots (Lanes 1–2, 3–4, and 5–6 are from line 1, 2 and 3, respectively)

**Fig. 5:**
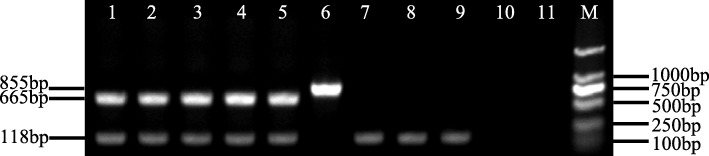
Transgenic hairy roots expressing *GUS* by RT-PCR. Lanes 1–5, independent transgenic root inoculated K599–1305; Lane 6, K599–1305; Lane 7–9, non-transgenic root inoculated K599–1305; Lane 10, K599; Lane 11, ddH_2_O

To determine whether the induced hairy roots are transgenic or not, we performed GUS (β- glucuronidase) staining and PCR analysis for each independent hairy root. Results showed that the PCR-positive roots were consistent with the GUS-positive ones (Fig. 4a-b). To further confirm the integration of the foreign DNA and its integrated site in the genome, TAIL-PCR [24] was performed. The results indicated that the foreign DNA was inserted into the soybean genome at different sites in the independent transgenic hairy roots (Fig. 4c, S1). In addition, RT-PCR analysis demonstrated that the foreign DNA was transcribed in the transgenic hairy roots (Fig. 5).

In the one-step transformation method, each seedling could produce transgenic positive roots where the transformation efficiency (defined as the percentage of the tested plant shoots that produced at least one transgenic root [16]) was 100% at 16 dpi (Table 1). The number of hairy roots and transgenic positive roots of each seedling were 5.80 ± 1.40 and 3.10 ± 0.96 at 16 dpi, and reached 12.4 ± 3.8 and 6.5 ± 2.7 at 20 dpi, respectively (Table 1). In contrast, in the two-step method, the transformation efficiency was only 40% at 16 dpi; the number of hairy roots and positive roots of each seedling were 1.43 ± 1.72 and 0.77 ± 0.97 at 16 dpi, and increased to 5.6 ± 1.8 and 1.8 ± 1.2 at 20 dpi, respectively (Table 1). Compared with two-step method, our one-step method resulted in four times of the transgenic hairy roots in each composite plant at 16 dpi (3.1 vs 0.77, Table 1). In conclusion, both the total number of hairy roots and transgene-positive roots of each composite plant by one-step method are higher than those of by the two-step method. Even if the transformation frequency (proportion of transgenic roots compared to total roots) were similar in the two transformation methods at 16 dpi (Table 1), the transformation frequency of one-step method was higher than that of the two-step transformation method at 20 dpi (Table 1). In the one-step method, the induced hairy roots grew faster than those of the two-step method (Table S1). Moreover, the one-step method can be applied to different genotypes of soybean tested, although some differences were observed among different genotypes in terms of transformation efficiency (Table 2).

**Table 1:** Comparison of transformation efficiency between one-step and two-step ARM transformation methods in Williams 82

Transformation method	dpi	Numbers of plants infected	Numbers of the explants with at least one hairy root	Numbers of hairy roots (per seedling)	Number of GUS-positive roots (per seedling)	Transformation efficiency	Transformation frequency
one-step	16	30	30	5.80 ± 1.40^A^	3.10 ± 0.96^A^	100%	53.45%
(Cutting)	20	30	30	12.4 ± 3.8 ^A^	6.5 ± 2.7 ^A^	100%	52.4%
two-step	16	30	14	1.43 ± 1.72^B^	0.77 ± 0.97^B^	40%	53.85%
(Stabbing)	20	30	24	5.6 ± 1.8^B^	1.8 ± 1.2^B^	66.7%	32.14%

Transformation root efficiency and frequency calculated at 16 dpi and 20 dpi. Letter A and B represented by different letters are very significantly different at the *p* = 0.01 value given. Values are mean ± SD for three independent replicates (*n* = 30 for each replicate)

**Table 2:** Transformation efficiency of the one-step ARM transformation method for different soybean ecotypes

Genotype	Numbers of plants infected	Numbers of the explants produced hairy roots	Numbers of hairy roots (per seedling)	Number of GUS-positive roots (per seedling)	Transformation efficiency	Transformation frequency
Williams82	30	30	12.4 ± 3.8 ^A^	6.5 ± 2.7 ^A^	100%	52.4%
Jindou 17	30	30	8.9 ± 2.0 ^CD^	3.4 ± 0.9 ^C^	96.7%	38.2%
Zhonghuang 39	32	32	8.7 ± 2.0 ^D^	3.1 ± 0.9 ^C^	96.7%	35.6%
Xiangfeng4	32	32	10.6 ± 2.2 ^BC^	3.8 ± 1.1 ^C^	100%	35.8%
Hedou19	30	30	10.0 ± 2.3 ^BCD^	3.9 ± 1.1 ^C^	100%	39%
PI377578	30	30	8.9 ± 2.1 ^CD^	3.1 ± 1.2 ^C^	96.7%	34.8%
Peking	30	30	11.5 ± 2.4 ^AB^	5.1 ± 1.3 ^B^	100%	44.3%

### The one-step ARM transformation method was used for analysis of soybean-rhizobia interaction

The leguminous plants, including soybean, can establish a symbiosis with rhizobia [25]. The legume-rhizobia interaction is highly specific, such that a particular genotype of soybean can nodulate with only a limited set of rhizobial strains. For example, the soybean genotype Williams 82 is resistant to rhizobial strain *S. fredii* USDA193 (Nod-) and the gene *Rfg1* in soybean is responsible for the nodulation restriction. If a loss of function mutation of *Rfg1* in Williams 82 happens, mature nitrogen-fixation nodules form on transgenic hairy roots inoculated with the USDA193 [21].

To evaluate the reliability of the one-step method, we used the one-step method to introduce a CRISPR/Cas9 vector targeting the *Rfg1* in Williams 82 (Nod-). Consistent with previous report, mature nitrogen-fixation nodules formed on the transformed hairy roots but not on the wild-type roots inoculated with the *S. fredii* USDA193 (Figure S2). The transgenic nodules were confirmed by PCR analyses and sequencing (Figure S3). Thus, our one-step transformation method is reliable and validated by soybean-rhizobia interaction analysis.

### Application of the one-step ARM transformation method to the promoter functional analysis and other plant species

**Fig. 6:**
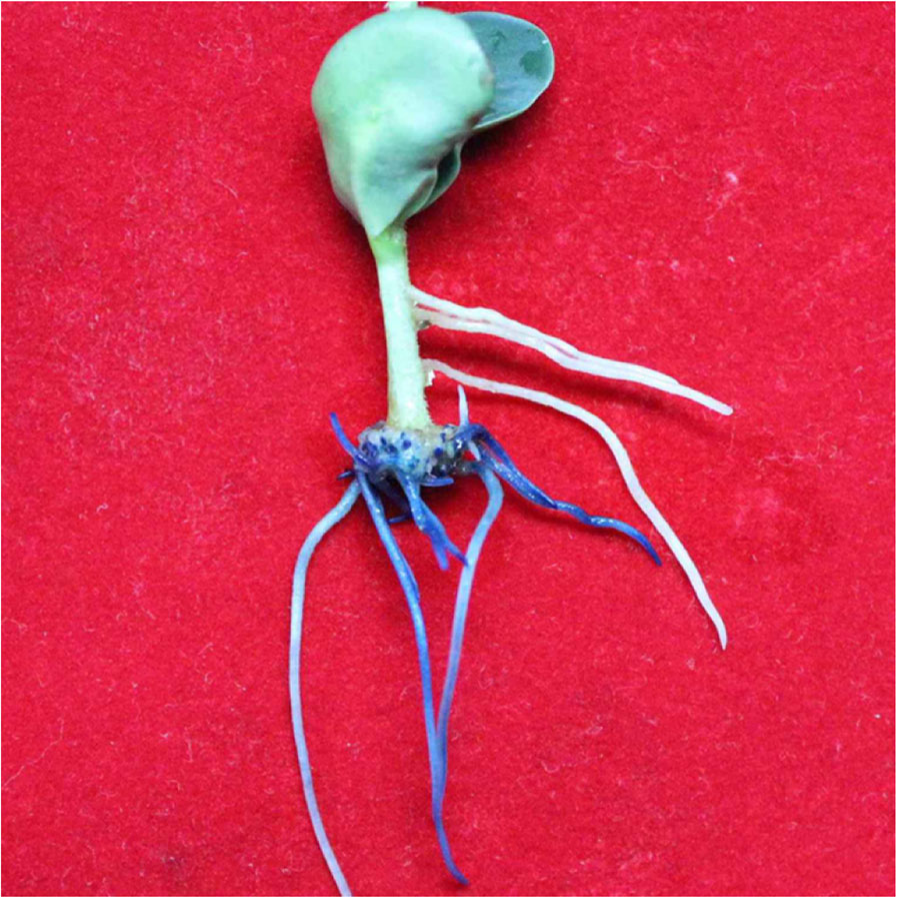
Histochemical localization of GUS activity in the p*YAO*:: *GUSPlus* soybean hairy roots

To test whether hairy roots are suitable for studying the promoter activities of genes, we used the one-step method to produce soybean transgenic hairy roots expressing *GUSPlus* gene driven by the *YAO* promoter from *Arabidopsis* [22]. Whole transformed pYAO::*GUSPlus* roots exhibited GUS signal and the root tips showed high level of activity (Fig. 6). This result is in consistent with that of *YAO* in *Arabidopsis* [22]. Therefore, we showed that the *Arabidopsis YAO* gene is conservatively expressed in soybean roots.

We also tested the one-step method whether can be applied to other plant species. Composite tomato plants have been successfully generated (Figure S4). Transformation efficiency and transformation frequency was 86.7 and 38.1%, respectively.

## Discussion

### One-step ARM transformation method is a faster, simpler, and more efficient for generating composite soybean plants with lower contamination risk

So far, ARM hairy roots transformation has already been established in diverse plant taxa covering more than 100 species [11, 13, 23, 26–28]. The development of composite plants is a critical milepost for functional analyses of candidate genes [19, 29–31]. However, as for the generation of composite plants, the one-step ARM transformation method was only reported in chickpea [32]. In chickpea, the apical stem section of 25–30 days old plant is used for inoculation with bacterial culture followed by hairy roots appeared after 15 dpi [32]. It takes 40–45 days to generate the chickpea hairy roots. In this study, the 7-day-old soybean seedlings were employed for inoculation. All infected explants produced hairy roots at 16 dpi, and the hairy root was 24.27 ± 8.76 cm (Fig. 3, Table S1). It takes only 23 days to induce the soybean hairy roots post-sowing. In contrast, in the traditional two-step ARM transformation in soybean, only 47% of the soybean seedlings produced hairy roots, and the hairy root was 0.53 ± 0.29 cm at 16 dpi (Fig. 3, Table S1). Therefore, our one-step protocol requires a shorter time for transformation.

In the two-step transformation method for soybean, the seedlings were injected by a syringe containing *A*. *rhizogenes* (HGCI) to induce the hairy roots [11, 23]. After the production of hairy roots, the primary root needs to be cut off, and the composite plants were transplanted into another new pot with sterile vermiculite [11, 23]. In our one-step transformation protocol, the composite plants can directly grow in sterile vermiculite after inoculation. All operations were finished from post-inoculation to growth of composite plants in the same pot. The two-step method might produce higher contamination risk in experiments for analyzing plant-microbe interactions due to more operation times and steps. Therefore, our one-step transformation protocol may reduce the contamination risk.

Higher transformation efficiency was achieved using the one-step method. In the two-step method, only 80% of the seedlings produced hairy roots, and the transformation efficiency was only 66.7% at 20 dpi (Table 1). In contrast, in our one-step method, all infected seedlings produced hairy roots, and the transformation efficiency was 100% (Table 1). Significantly, both total number of hairy roots and transgene-positive roots of each composite plant are higher than those of two-step method (Table 1). The possible reason for high transformation efficiency is that the slant cut of hypocotyl with larger surface area may increase the possibility of *A*. *rhizogenesis* infection. In addition, in our one-step method, the hairy roots appeared earlier and grew faster than that of the two-step method (Table S1); the possible reasons might be that the hairy roots in the one-step protocol grow in the dark soil and absorb more mineral nutrients and water. Furthermore, the induced hairy roots might grow fast without the inhibition of primary root.

Thus far, in traditional two-step ARM transformation method, besides composite soybean plants produced by stabbing the hypocotyl, Ex-vitro (or In-vitro) ARM transformation method was also reported in the two papers [14, 16]. However, in the Ex-vitro (or In-vitro) transformation method, the hairy roots are induced on Fibrgro® cubes, or in rockwool [14, 16]. It is tedious, labor-intensive, and time-consuming to detach the hairy roots from the medium. In addition, this procedure might result in some induced hairy roots and/or root hairs broken or damaged. It can also increases the contamination risk in the study on plant-microbe interactions. In contrast, our one-step protocol sidesteps the tedious work of detaching the roots from medium.

In both one-step and two-step methods, not 100% generated hairy roots from a composite plant are transgenic. Non-transgenic roots needn’t be cut off and can play a part in a negative control for the wild-type phenotype compared with the transgene-positive roots. Based on our results and previous discussion, our one-step transformation protocol is a particularly optimized method, which can serve as parsing candidate gene functions in root-microbe interactions, such as symbiotic interactions including *Rhizobium* symbiosis in legume species, e.g. soybean, pea and *Medicago truncatula* and plant-nematode interactions.

### Applicability of the one-step ARM transformation method in studying promoter function and other species

ARM hairy root transformation has been used for various purposes, such as plant-microbe interactions (rhizobia, arbuscular mycorrhizal fungi, pathogens etc.), root-shoot communications involved in plant hormones, RNAs and proteins, and plant-environment interactions (biotic/abiotic stresses) [11, 23, 26–28, 33]. In this study, we applied the new one-step method to analyze the function of *Arabidopsis YAO* promoter in soybean roots. The result indicated that the expression pattern of *Arabidopsis YAO* is conserved in soybean roots [22].

In addition to experiments in soybean, we also tested the one-step transformation method whether can be applied to other plant species. Composite tomato plants have been successfully generated (Figure S4). These results suggest that the new method can apply to other plant species. For a given plant species, it needs to screen for a compatible and high-efficiency *A*. *rhizogenesis* strain because co-transformation efficiency varies for different *A*. *rhizogenesis* strains [34]*.*

### Some key steps influence the transformation efficiency of the one-step ARM transformation method

To achieve the high transformation efficiency, several key steps need to pay more attention. Seedling age is important for high transformation efficiency. We recommend seven-day-old healthy seedlings used, and the poorly developed seedlings should be discarded. The seedling should be cut diagonally in the liquid of *A*. *rhizogenes* (HGCI) using sharp scalpel. Slanting cut can increase the surface area for optimum bacterial infection. *A*. *rhizogenes* should be freshly cultured and healthy. High humidity is required to be maintained and inoculated seedlings do not need to be watered during the induction of hairy roots. After generation of hairy roots, the humidity should be slowly decreased by removing the transparent lid or plastic bag (opening the ventilation holes or making some holes on the lid or bags) after 16 dpi to make composite plants adapt to the environment well.

## Conclusions

A new, faster, simpler, and more efficient one-step ARM transformation method for soybean with lower contamination risk is established, which is especially optimal protocol for studying on soybean-rhizobia interactions. We anticipate the new transformation method will be widely used for analysis of root-related events in tomato and other plants besides soybean.

## Methods

### Plant materials and growth conditions

Soybean seeds were surface-sterilized by 3 min treatment in a 70% ethanol, rinsed five times with sterile water and planted into a pot with sterile wet vermiculite (11.6 cm × 8 cm × 9.5 cm) at a depth of 1–2 cm (0.25× Gamborg B-5 basal medium). Plants were grown in a greenhouse under 80 μmol m^− 2^ s^− 1^ for 16 h light /8 h dark condition, at 24–26 °C, approximately 70% relative humidity. Soybean seeds of genotypes Williams 82, Jindou 17, Zhonghuang 39, Xiangfeng4, Hedou19, PI377578, and Peking were provided by the National Key Facility for Crop Gene Resources and Genetic Improvement (NFCIR), Institute of Crop Science, Chinese Academy of Agricultural Sciences.

### Construction of CRISPR/Cas9 construct targeting *Rfg1* gene and *YAO* promoter::*GUSPlus* vector and *A. rhizogenes* strain

CRISPR/Cas9-mediated *Rfg1* gene knockout vector was constructed as described previously with the following modification [21]. *GUS* reporter gene driven by CaMV 35S promoter replaced the *Hpt II* (*Hygromycin Phosphotransferase II*), which can be used for selection of transgenic positive root from a composite plant. One target site was designed to knockout the *Rfg1*. The CRISPR/Cas9 construct was named as pGSE401-KtRfg1 (Figure S5a). The *YAO* promoter sequence was amplified by PCR from *Arabidopsis thaliana* (ecotype Col-0) genomic DNA using primers YAOF18 with a *Kpn*I restriction site and YAO19R with an *Nco*I restriction site. The digested PCR products using restriction enzymes *Kpn*I and *Nco*I were cloned into pCAMBIA1305 vector, which placed *YAO* promoter to drive the transcription of the *GUSPlus*. The binary vector of pYAO::*GUSPlus* was termed as pYGUS1305. All constructs were verified by sequencing. The maps of all constructs and sequences are shown in Figure S5. The binary vector pGSE401-KtRfg1 (Figure S5a), pCAMBIA1305 (Figure S5b) and pYGUS1305 (Figure S5c) were introduced into *A. rhizogenes* strain K599 [34, 35] by electroporation, respectively. K599 strain was cultured as described previously [35]. To make the inoculation medium, fresh culture K599 bacteria (HGCI) grown on the plate was re-suspended using 0.25× Gamborg B-5 basal medium to OD600 ≈ 0.6–1.0. Gamborg B-5 basal medium was purchased in powder form from Phyto Technology laboratories (Shawnee Mission, Kansas). Nucleotide sequences of all primers used in this paper are shown in Table S2.

### One-step ARM hairy root transformation and generation of composite soybean plants

In the one-step protocol by cutting the hypocotyl, the primary root was excised from 7-day-old healthy seedlings with unfolded true leaves using a pair of sterile scissors. 0.7–1 cm apical portion of the hypocotyl maintained was cut diagonally (0.5 cm cut) with a sterile scalpel in the inoculation medium. The slant cut of partial hypocotyl left was scraped on the plate grown K599-HGCI and then directly planted into the pot with completely wet sterile vermiculite, which was watered with autoclaved distilled water prior to use. And then each seedling was inoculated with ~ 5 ml the inoculation medium. High humidity (covering with a sterile plastic bag) in seedlings grown environment was maintained after the cut of the hypocotyl was inoculated and infected with K559-HGCI (key steps of the procedure is shown in Fig. 1a-e). Do not need to water the inoculated seedlings during the following 2 weeks. After 2 weeks, the hairy roots developed. The humidity was slowly decreased by making the ventilation holes on the transparent sterile plastic bag gradually over 2 days. After that, the bag was removed. Plants were regularly checked for maintaining water (0.25× Gamborg B-5) and humidity. The two-step transformation method by stabbing the hypocotyl was carried out as described previously [11].

### Rhizobium growth conditions and the nodulation assay

*S. fredii* USDA193 culture and nodulation assay were performed according to Fan et al. [21]. Each seedling at 16 dpi was water-inoculated with cultured rhizobia. The sterile N-free nutrient solution [11] was used for watering the plants.

### Histochemical staining of GUS, DNA extraction, PCR (polymerase chain reaction), and TAIL-PCR (thermal asymmetric interlaced PCR) analysis

There is a *GUSPlus* gene in the pCAMBIA1305.1 (Figure S5b), thus, GUS activity assay was carried out. Histochemical staining of GUS activity qualitative assay was conducted as described [36] with minor modifications. The transformed hairy roots were immersed in GUS staining solution using 1 mM potassium ferricyanide and 1 mM potassium ferrocyanide instead of 8 mM mercaptoethanol at 37 °C for 2–10 h. The stained samples were rinsed in a 70% ethanol for 10–20 min. To further identify the transgenic and non-transgenic roots, PCR assay was also performed to amplify the *GUSPlus*. To determine the CRISPR/Cas9-mediated gene indels in the *Rfg1*, we extracted the genomic DNA of the nodules followed by PCR amplification, and DNA sequencing. For PCR analysis, genomic DNA was isolated from leaves or nodule as described previously [37]. PCR amplification was performed as published previously [38]. For CRISPR/Cas9-mediated gene disrupt, PCR product was validated the targeted gene disruption by DNA sequencing. The PCR product was subjected to Sanger sequencing. The TAIL-PCR was performed according to Tan et al [24] with the following modifications. Pre-amplification of transgenic hairy roots using primers pooled LADs (included LAD1–1, LAD1–2, LAD1–3 and LAD1–4) and SPNos1. Then the primary TAIL-PCR was performed using the nested specific primer SPNos2 and AC. Secondary TAIL-PCR products were diluted 500-fold and used as templates to perform the third amplification with nested primers SPNos3 and AC. The expected differential sizes of PCR product from the secondary and tertiary TAIL-PCR are 111 bp. The Primers SPNos1, SPNos2 and SPNos3 are specific to pCAMBIA1305 binary vector having the *Nos* terminator sequence adjacent to RB of T-DNA.

### RT-PCR analysis

RT-PCR was performed to detect the transcript of *GUSPlus*. The sizes of PCR and RT-PCR products (855 bp and 665 bp, respectively) are different because the amplification fragment covers the intron region of *GUSPlus*. The total RNA was isolated, poly (A)^±^ mRNA was purified from total RNA, first-strand cDNA was synthesized, and RT-PCR amplification were carried out as previously described [38] with the following modifications. RT-PCR reaction was conducted in a reaction mixture to amplify the transcripts of *GmActin* and *GUSPlus*. Reaction conditions: 3 min at 96 °C; 30 cycles (30 s at 95 °C, 45 s at 56 °C, 1 min at 72 °C); and 5 min at 72 °C. The soybean *Gmactin* gene was used as the endogenous control (118 bp product). The gene-specific primer pairs RTGusF and RTGusR for *GUSPlus* gene, and the primer pairs GmActinF and GmActinR for *Gmactin* gene were used.

### The root length analysis

The root systems in each treatment were scanned using an EPSON V700 Scanner (EPSON (China) Co., Ltd), and the root morphological indexes root length was analyzed with WinRHIZO PRO 2012 Root Analysis System (Regent Instruments Inc., Quebec, Canada).

### Statistical analysis

Each experiment with 30 seedlings was a biological replicate and repeated two additional times. Data were analyzed using Excel and DPS statistical software. Each value represents the mean of three independent experiments with standard deviation (SD). a, b followed by different letters indicate significant difference at 5% level of significance by LSD tests in Fig. 2B.

## Supplementary information

**Additional file 1: Figure S1.** The T-DNA insertion sites in the soybean genome determined by sequencing of TAIL-PCR products. Three examples of sequencing analysis (partial sequences are indicated) of the TAIL-PCR products from three different independent transgenic hairy roots (in A, B, and C, respectively). Chr. 13 (in panel A), Chr. 20 (in panel B), Chr. 2 (in panel C) represented chromosome number. The gray filled triangles indicated the T-DNA insertions and the numbers showed the positions of the insertion site. Green highlighted letters indicated the T-DNA sequences of the right border side from pCAMBIA1305.1. Yellow highlighted letters indicated genomic flanking sequences of T-DNA insertion.

**Additional file 2: Figure S2.** CRISPR/Cas9-mediated knockout of *Rfg1* in the Williams 82 (Nod-). Mature fixation nitrogen root nodules formed on a transgenic root (pointed out by arrows)

**Additional file 3: Figure S3.** A sequencing identification from CRISPR/Cas9-mediated knockout of *Rfg1* in the Williams 82 (Nod–) background. One targeted knockout site (indicated in the black box) in Williams 82 wild type background and its flanking sequence. The protospacer adjacent motif (PAM) is ‘TGG’ (a). Sequencing analysis of the DNA from transgenic nodules revealed that two mutant alleles were caused, one with a 152-bp deletion (b) and another with a 70- bp deletion (c).

**Additional file 4: Figure S4.** One-step generation of composite tomato plant with transgenic roots by ARM transformation. Histochemical analyses of tomato hairy roots transformed with K599 harboring pCAMBIA1305 where contains GUS reporter gene driven by CaMV35S promoter. Tomato (*Solanum lycopersicum*) seeds Maofen 802 were used.

**Additional file 5: Figure S5.** Maps and sequences of the binary vector. pGSE401-KtRfg1vector. The targeted site was highlighted in the red background (a); pCAMBIA1305 vector (b); pYGUS1305 (c).

**Additional file 6: Figure S6.** The original blot presented in Fig. 4b.

**Additional file 7: Figure S7.** The original blot presented in Fig. 4c.

**Additional file 8: Figure S8.** The original blot presented in Fig. 5.

**Additional file 9: Table S1.** A comparison of hairy roots produced by one-step and two-step method.

**Additional file 10: Table S2.** All primers sequences used in this paper.

## Data Availability

The data generated or analyzed in this study are included in this article and its supplementary information files. Other materials that support the findings of this study are available from the corresponding author on request.

## Abbreviations

ARM: *Agrobacterium rhizogenes*-mediated
GWAS: Genome-wide association studies
CRISPR/Cas9: Clustered regularly interspaced short palindromic repeats-associated Cas9
HGCI: Harboring the gene construct of interest
ALRs: Adventitious laterals roots
dpi: Days post-inoculation
GUS: β- glucuronidase
PCR: Polymerase chain reaction
*Hpt II*: (*Hygromycin Phosphotransferase II*)
TAIL-PCR: Thermal asymmetric interlaced PCR
RT-PCR: Reverse transcriptase polymerase chain reaction
K599–1305: K599-harboring pCAMBIA1305 vector
