# Supplementary figures and images for: One-step generation of composite soybean plants with transgenic roots by Agrobacterium rhizogenes-mediated transformation
Source: BMC Plant Biol. 2020 May 12;20:208. doi: 10.1186/s12870-020-02421-4 (PMC7333419; doi:10.1186/s12870-020-02421-4)

**A**

T-DNA insert

Chr. 13

17652626

17652645

**
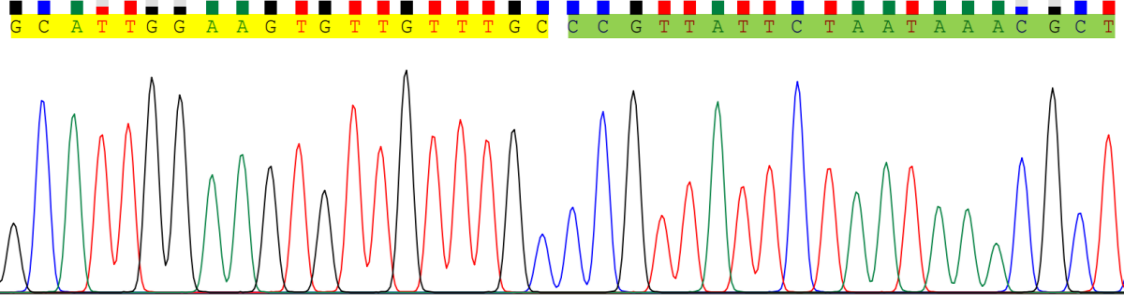
**

**B**

Chr. 20

38040330

38040349

**
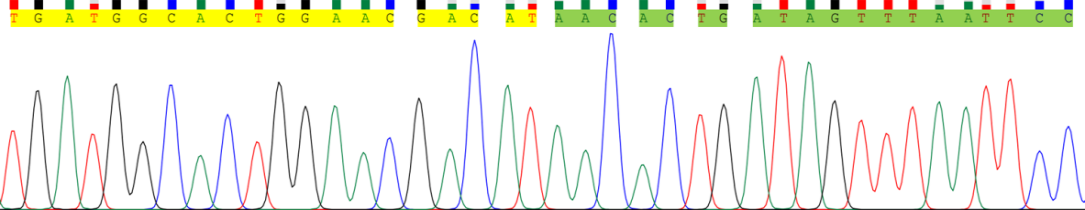
**

**C**

Chr. 02

6632296

6632315

**
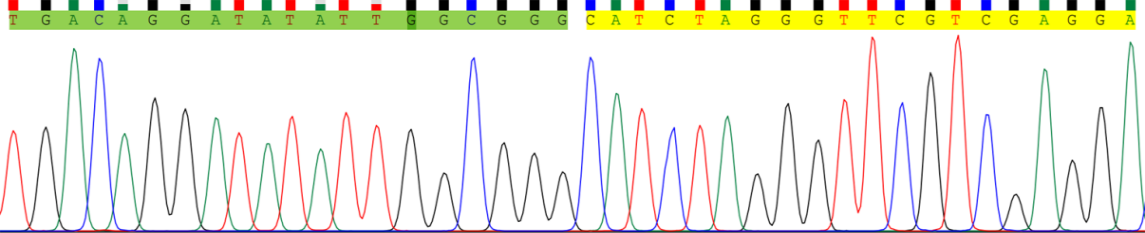
**

Supplement: Supplementary file 1 — Additional file 1: Figure S1. The T-DNA insertion sites in the soybean genome determined by sequencing of TAIL-PCR products. Three examples of sequencing analysis (partial sequences are indicated) of the TAIL-PCR products from three different independent transgenic hairy roots (in A, B, and C, respectively). Chr. 13 (in panel A), Chr. 20 (in panel B), Chr. 2 (in panel C) represented chromosome number. The gray filled triangles indicated the T-DNA insertions and the numbers showed the positions of the insertion site. Green highlighted letters indicated the T-DNA sequences of the right border side from pCAMBIA1305.1. Yellow highlighted letters indicated genomic flanking sequences of T-DNA insertion. [file 12870_2020_2421_MOESM1_ESM.docx]

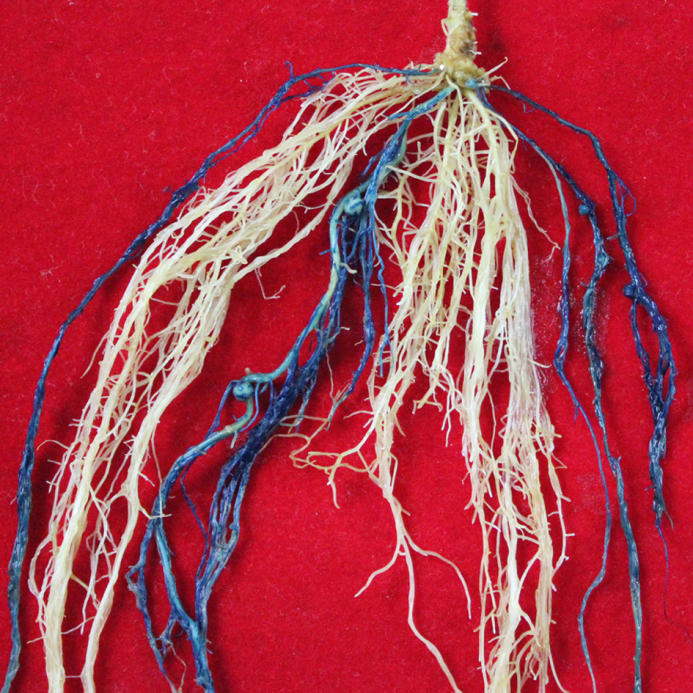

Supplement: Supplementary file 2 — Additional file 2: Figure S2. CRISPR/Cas9-mediated knockout of Rfg1 in the Williams 82 (Nod-). Mature fixation nitrogen root nodules formed on a transgenic root (pointed out by arrows) [file 12870_2020_2421_MOESM2_ESM.docx]

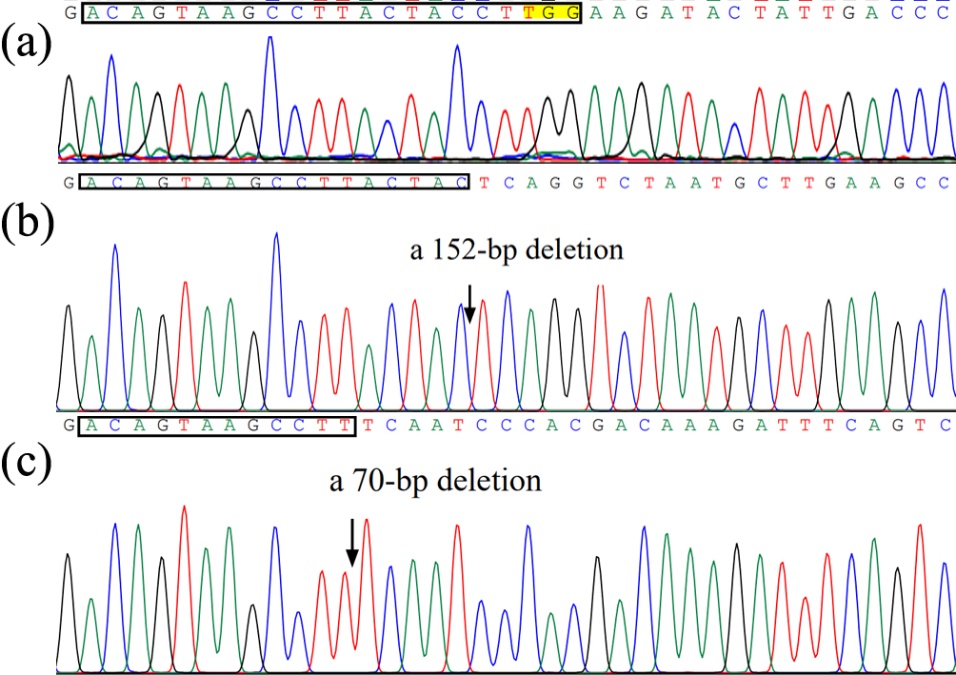

Supplement: Supplementary file 3 — Additional file 3: Figure S3. A sequencing identification from CRISPR/Cas9-mediated knockout of Rfg1 in the Williams 82 (Nod–) background. One targeted knockout site (indicated in the black box) in Williams 82 wild type background and its flanking sequence. The protospacer adjacent motif (PAM) is ‘TGG’ (a). Sequencing analysis of the DNA from transgenic nodules revealed that two mutant alleles were caused, one with a 152-bp deletion (b) and another with a 70- bp deletion (c). [file 12870_2020_2421_MOESM3_ESM.docx]

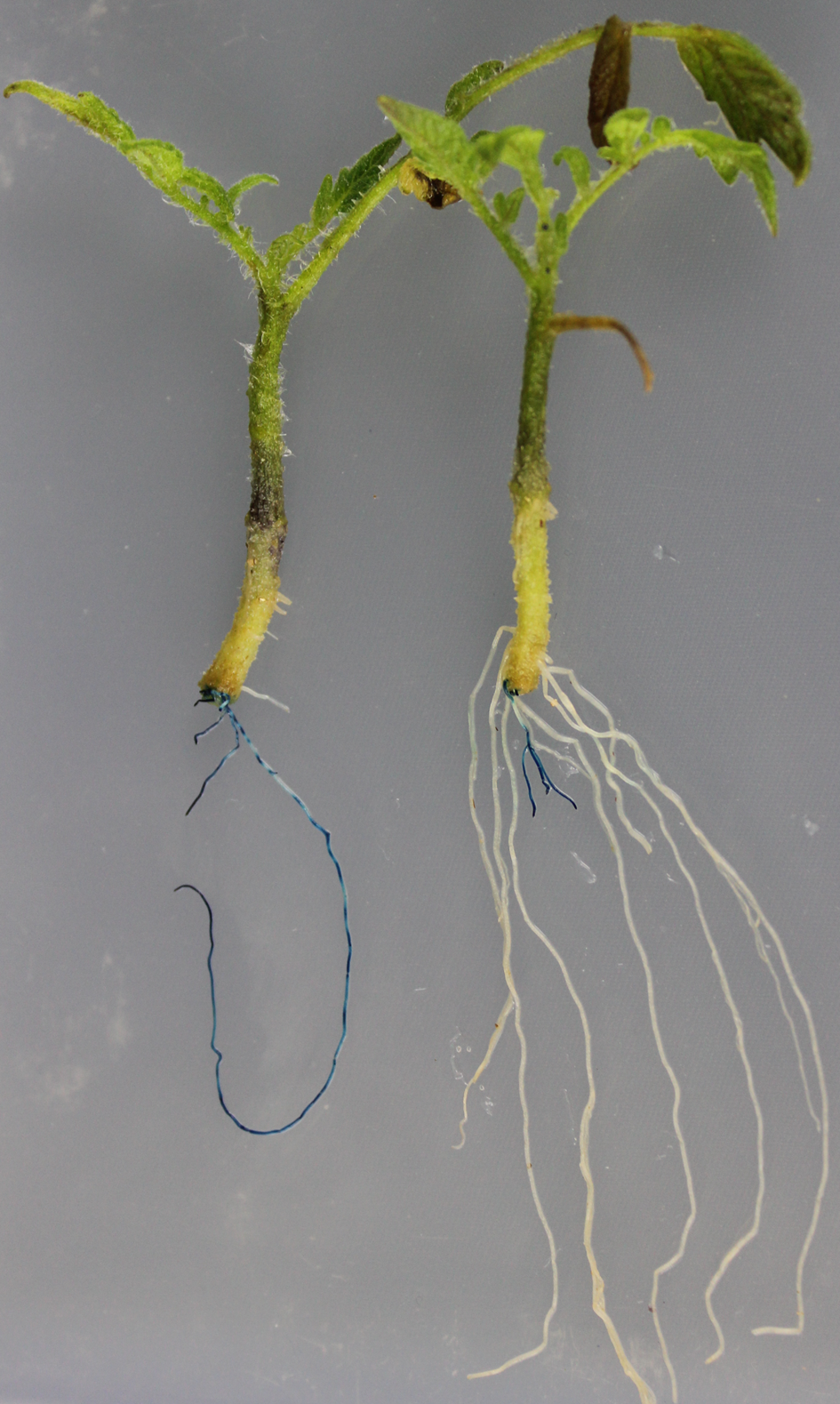

Supplement: Supplementary file 4 — Additional file 4: Figure S4. One-step generation of composite tomato plant with transgenic roots by ARM transformation. Histochemical analyses of tomato hairy roots transformed with K599 harboring pCAMBIA1305 where contains GUS reporter gene driven by CaMV35S promoter. Tomato (Solanum lycopersicum) seeds Maofen 802 were used. [file 12870_2020_2421_MOESM4_ESM.docx]

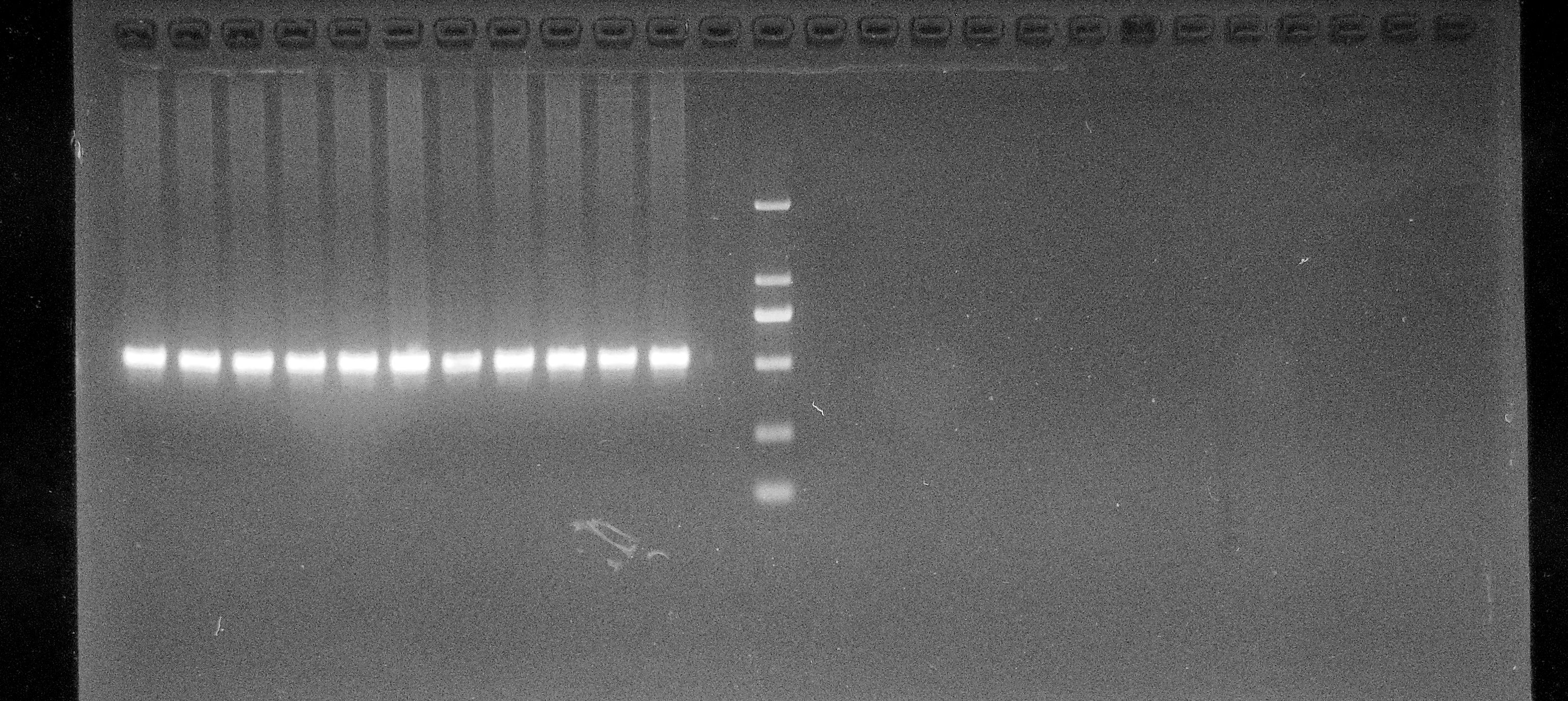

Supplement: Supplementary file 6 — Additional file 6: Figure S6. The original blot presented in Fig. 4b. [file 12870_2020_2421_MOESM6_ESM.jpg]

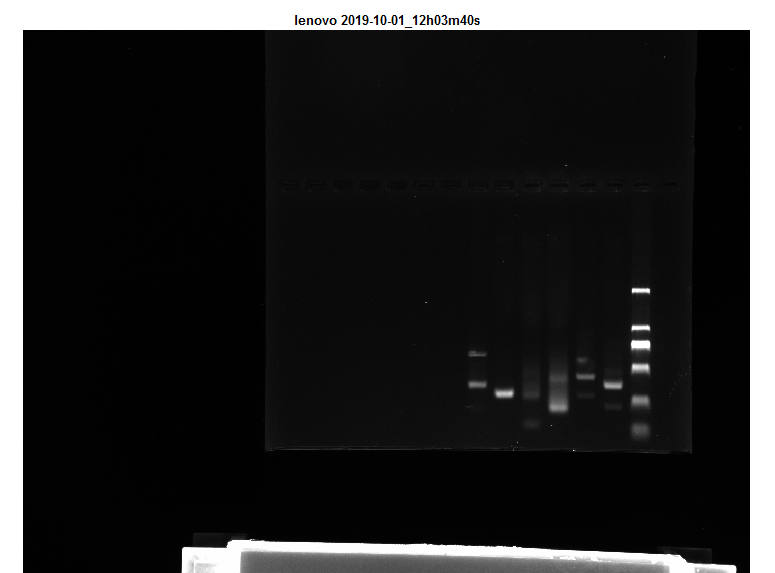

Supplement: Supplementary file 7 — Additional file 7: Figure S7. The original blot presented in Fig. 4c. [file 12870_2020_2421_MOESM7_ESM.tif]

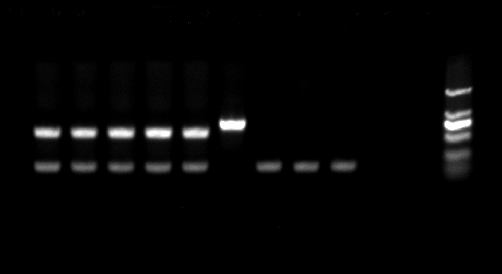

Supplement: Supplementary file 8 — Additional file 8: Figure S8. The original blot presented in Fig. 5. [file 12870_2020_2421_MOESM8_ESM.tif]
