# Supplementary material for: One-step generation of composite soybean plants with transgenic roots by Agrobacterium rhizogenes-mediated transformation
Source: BMC Plant Biol. 2020 May 12;20:208. doi: 10.1186/s12870-020-02421-4 (PMC7333419; doi:10.1186/s12870-020-02421-4)
Supplement: Supplementary file 5 — Additional file 5: Figure S5. Maps and sequences of the binary vector. pGSE401-KtRfg1vector. The targeted site was highlighted in the red background (a); pCAMBIA1305 vector (b); pYGUS1305 (c). [file 12870_2020_2421_MOESM5_ESM.docx]

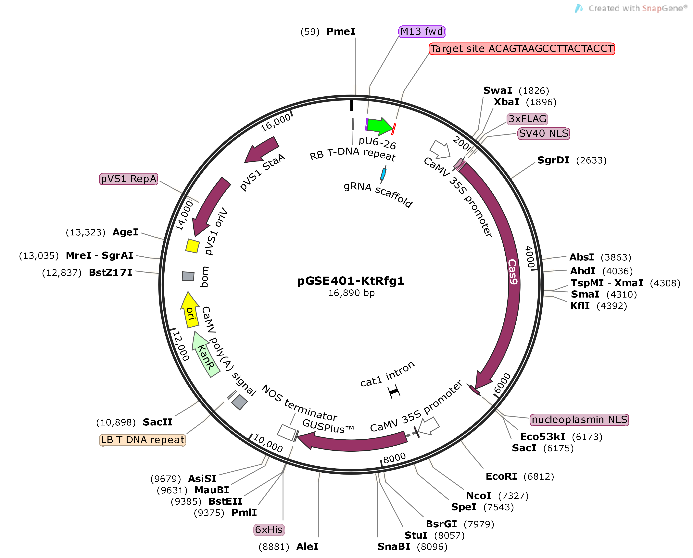


(a)


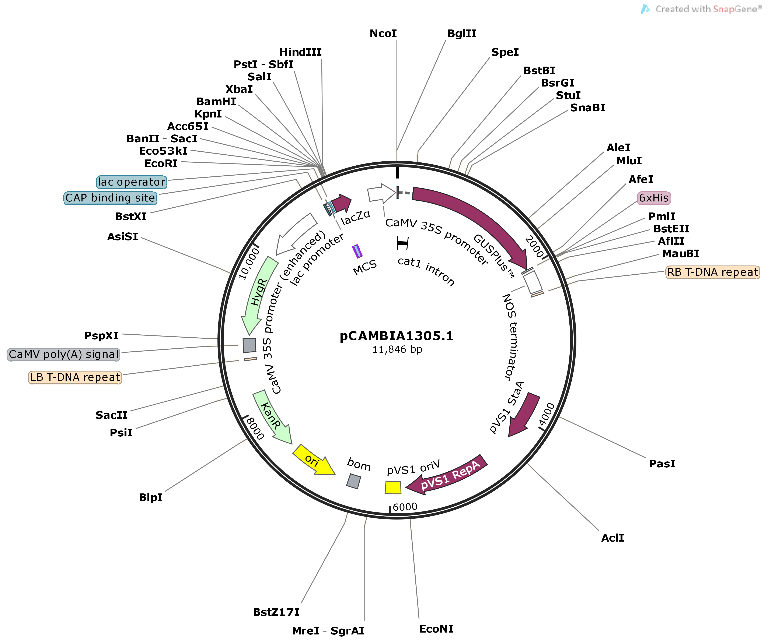
(b)


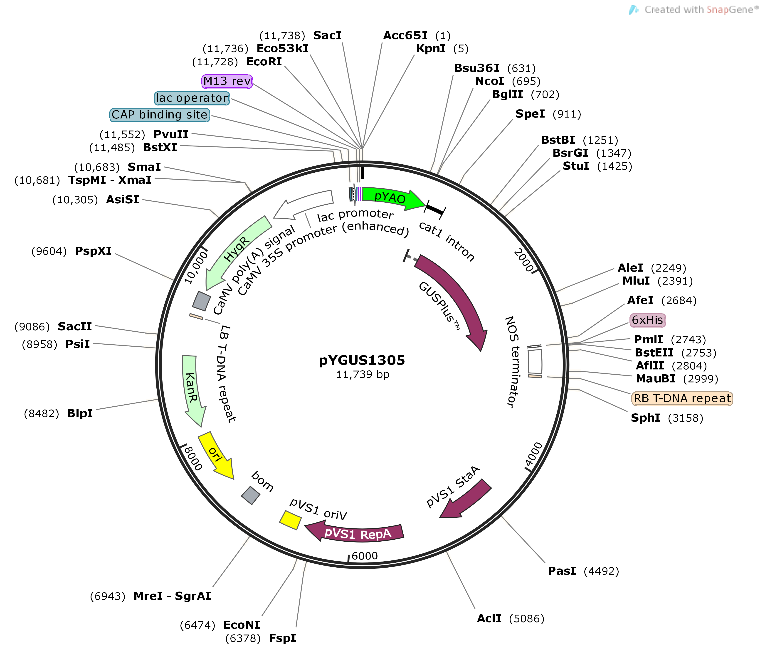
(c)

>pGSE401-KtRfg1, complete sequence, 16890bp

TaaacgctcttttctcttaggtttacccgccaatatatcctgtcaaacactgatagtttaaactgaaggcgggaaacgacaatctgatccaagctcaagctgctctagcattcgccattcaggctgcgcaactgttgggaagggcgatcggtgcgggcctcttcgctattacgccagctggcgaaagggggatgtgctgcaaggcgattaagttgggtaacgccagggttttcccagtcacgacgttgtaaaacgacggccagtgccaagcttcgacttgccttccgcacaatacatcatttcttcttagctttttttcttcttcttcgttcatacagtttttttttgtttatcagcttacattttcttgaaccgtagctttcgttttcttctttttaactttccattcggagtttttgtatcttgtttcatagtttgtcccaggattagaatgattaggcatcgaaccttcaagaatttgattgaataaaacatcttcattcttaagatatgaagataatcttcaaaaggcccctgggaatctgaaagaagagaagcaggcccatttatatgggaaagaacaatagtatttcttatataggcccatttaagttgaaaacaatcttcaaaagtcccacatcgcttagataagaaaacgaagctgagtttatatacagctagagtcgaagtagtgattgACAGTAAGCCTTACTACCTgttttagagctagaaatagcaagttaaaataaggctagtccgttatcaacttgaaaaagtggcaccgagtcggtgcttttttttgcaaaattttccagatcgatttcttcttcctctgttcttcggcgttcaatttctggggttttctcttcgttttctgtaactgaaacctaaaatttgacctaaaaaaaatctcaaataatatgattcagtggttttgtacttttcagttagttgagttttgcagttccgatgagataaaccaataagcttgcatgcctgcaggtcaacatggtggagcacgacacacttgtctactccaaaaatatcaaagatacagtctcagaagaccaaagggcaattgagacttttcaacaaagggtaatatccggaaacctcctcggattccattgcccagctatctgtcactttattgtgaagatagtggaaaaggaaggtggctcctacaaatgccatcattgcgataaaggaaaggccatcgttgaagatgcctctgccgacagtggtcccaaagatggacccccacccacgaggagcatcgtggaaaaagaagacgttccaaccacgtcttcaaagcaagtggattgatgtgataacatggtggagcacgacacacttgtctactccaaaaatatcaaagatacagtctcagaagaccaaagggcaattgagacttttcaacaaagggtaatatccggaaacctcctcggattccattgcccagctatctgtcactttattgtgaagatagtggaaaaggaaggtggctcctacaaatgccatcattgcgataaaggaaaggccatcgttgaagatgcctctgccgacagtggtcccaaagatggacccccacccacgaggagcatcgtggaaaaagaagacgttccaaccacgtcttcaaagcaagtggattgatgtgatatctccactgacgtaagggatgacgcacaatcccactatccttcgcaagacccttcctctatataaggaagttcatttcatttggagaggacctcgacctcaacacaacatatacaaaacaaacgaatctcaagcaatcaagcattctacttctattgcagcaatttaaatcatttcttttaaagcaaaagcaattttctgaaaattttcaccatttacgaacgatactcgagtaatctagatggattacaaggaccacgacggggattacaaggaccacgacattgattacaaggatgatgatgacaagatggctccgaagaagaagaggaaggttggcatccacggggtgccagctgctgacaagaagtactcgatcggcctcgatattgggactaactctgttggctgggccgtgatcaccgacgagtacaaggtgccctcaaagaagttcaaggtcctgggcaacaccgatcggcattccatcaagaagaatctcattggcgctctcctgttcgacagcggcgagacggctgaggctacgcggctcaagcgcaccgcccgcaggcggtacacgcgcaggaagaatcgcatctgctacctgcaggagattttctccaacgagatggcgaaggttgacgattctttcttccacaggctggaggagtcattcctcgtggaggaggataagaagcacgagcggcatccaatcttcggcaacattgtcgacgaggttgcctaccacgagaagtaccctacgatctaccatctgcggaagaagctcgtggactccacagataaggcggacctccgcctgatctacctcgctctggcccacatgattaagttcaggggccatttcctgatcgagggggatctcaacccggacaatagcgatgttgacaagctgttcatccagctcgtgcagacgtacaaccagctcttcgaggagaaccccattaatgcgtcaggcgtcgacgcgaaggctatcctgtccgctaggctctcgaagtctcggcgcctcgagaacctgatcgcccagctgccgggcgagaagaagaacggcctgttcgggaatctcattgcgctcagcctggggctcacgcccaacttcaagtcgaatttcgatctcgctgaggacgccaagctgcagctctccaaggacacatacgacgatgacctggataacctcctggcccagatcggcgatcagtacgcggacctgttcctcgctgccaagaatctgtcggacgccatcctcctgtctgatattctcagggtgaacaccgagattacgaaggctccgctctcagcctccatgatcaagcgctacgacgagcaccatcaggatctgaccctcctgaaggcgctggtcaggcagcagctccccgagaagtacaaggagatcttcttcgatcagtcgaagaacggctacgctgggtacattgacggcggggcctctcaggaggagttctacaagttcatcaagccgattctggagaagatggacggcacggaggagctgctggtgaagctcaatcgcgaggacctcctgaggaagcagcggacattcgataacggcagcatcccacaccagattcatctcggggagctgcacgctatcctgaggaggcaggaggacttctaccctttcctcaaggataaccgcgagaagatcgagaagattctgactttcaggatcccgtactacgtcggcccactcgctaggggcaactcccgcttcgcttggatgacccgcaagtcagaggagacgatcacgccgtggaacttcgaggaggtggtcgacaagggcgctagcgctcagtcgttcatcgagaggatgacgaatttcgacaagaacctgccaaatgagaaggtgctccctaagcactcgctcctgtacgagtacttcacagtctacaacgagctgactaaggtgaagtatgtgaccgagggcatgaggaagccggctttcctgtctggggagcagaagaaggccatcgtggacctcctgttcaagaccaaccggaaggtcacggttaagcagctcaaggaggactacttcaagaagattgagtgcttcgattcggtcgagatctctggcgttgaggaccgcttcaacgcctccctggggacctaccacgatctcctgaagatcattaaggataaggacttcctggacaacgaggagaatgaggatatcctcgaggacattgtgctgacactcactctgttcgaggaccgggagatgatcgaggagcgcctgaagacttacgcccatctcttcgatgacaaggtcatgaagcagctcaagaggaggaggtacaccggctgggggaggctgagcaggaagctcatcaacggcattcgggacaagcagtccgggaagacgatcctcgacttcctgaagagcgatggcttcgcgaaccgcaatttcatgcagctgattcacgatgacagcctcacattcaaggaggatatccagaaggctcaggtgagcggccagggggactcgctgcacgagcatatcgcgaacctcgctggctcgccagctatcaagaaggggattctgcagaccgtgaaggttgtggacgagctggtgaaggtcatgggcaggcacaagcctgagaacatcgtcattgagatggcccgggagaatcagaccacgcagaagggccagaagaactcacgcgagaggatgaagaggatcgaggagggcattaaggagctggggtcccagatcctcaaggagcacccggtggagaacacgcagctgcagaatgagaagctctacctgtactacctccagaatggccgcgatatgtatgtggaccaggagctggatattaacaggctcagcgattacgacgtcgatcatatcgttccacagtcattcctgaaggatgactccattgacaacaaggtcctcaccaggtcggacaagaaccggggcaagtctgataatgttccttcagaggaggtcgttaagaagatgaagaactactggcgccagctcctgaatgccaagctgatcacgcagcggaagttcgataacctcacaaaggctgagaggggcgggctctctgagctggacaaggcgggcttcatcaagaggcagctggtcgagacacggcagatcactaagcacgttgcgcagattctcgactcacggatgaacactaagtacgatgagaatgacaagctgatccgcgaggtgaaggtcatcaccctgaagtcaaagctcgtctccgacttcaggaaggatttccagttctacaaggttcgggagatcaacaattaccaccatgcccatgacgcgtacctgaacgcggtggtcggcacagctctgatcaagaagtacccaaagctcgagagcgagttcgtgtacggggactacaaggtttacgatgtgaggaagatgatcgccaagtcggagcaggagattggcaaggctaccgccaagtacttcttctactctaacattatgaatttcttcaagacagagatcactctggccaatggcgagatccggaagcgccccctcatcgagacgaacggcgagacgggggagatcgtgtgggacaagggcagggatttcgcgaccgtcaggaaggttctctccatgccacaagtgaatatcgtcaagaagacagaggtccagactggcgggttctctaaggagtcaattctgcctaagcggaacagcgacaagctcatcgcccgcaagaaggactgggatccgaagaagtacggcgggttcgacagccccactgtggcctactcggtcctggttgtggcgaaggttgagaagggcaagtccaagaagctcaagagcgtgaaggagctgctggggatcacgattatggagcgctccagcttcgagaagaacccgatcgatttcctggaggcgaagggctacaaggaggtgaagaaggacctgatcattaagctccccaagtactcactcttcgagctggagaacggcaggaagcggatgctggcttccgctggcgagctgcagaaggggaacgagctggctctgccgtccaagtatgtgaacttcctctacctggcctcccactacgagaagctcaagggcagccccgaggacaacgagcagaagcagctgttcgtcgagcagcacaagcattacctcgacgagatcattgagcagatttccgagttctccaagcgcgtgatcctggccgacgcgaatctggataaggtcctctccgcgtacaacaagcaccgcgacaagccaatcagggagcaggctgagaatatcattcatctcttcaccctgacgaacctcggcgcccctgctgctttcaagtacttcgacacaactatcgatcgcaagaggtacacaagcactaaggaggtcctggacgcgaccctcatccaccagtcgattaccggcctctacgagacgcgcatcgacctgtctcagctcgggggcgacaagcggccagcggcgacgaagaaggcggggcaggcgaagaagaagaagtgagctcagagctttcgttcgtatcatcggtttcgacaacgttcgtcaagttcaatgcatcagtttcattgcgcacacaccagaatcctactgagtttgagtattatggcattgggaaaactgtttttcttgtaccatttgttgtgcttgtaatttactgtgttttttattcggttttcgctatcgaactgtgaaatggaaatggatggagaagagttaatgaatgatatggtccttttgttcattctcaaattaatattatttgttttttctcttatttgttgtgtgttgaatttgaaattataagagatatgcaaacattttgttttgagtaaaaatgtgtcaaatcgtggcctctaatgaccgaagttaatatgaggagtaaaacacttgtagttgtaccattatgcttattcactaggcaacaaatatattttcagacctagaaaagctgcaaatgttactgaatacaagtatgtcctcttgtgttttagacatttatgaactttcctttatgtaattttccagaatccttgtcagattctaatcattgctttataattatagttatactcatggatttgtagttgagtatgaaaatattttttaatgcattttatgacttgccaattgattgacaacGAATTCGAACTCGCCGTAAAGACTGGCGAACAGTTCATACAGAGTCTCTTACGACTCAATGACAAGAAGAAAATCTTCGTCAACATGGTGGAGCACGACACACTTGTCTACTCCAAAAATATCAAAGATACAGTCTCAGAAGACCAAAGGGCAATTGAGACTTTTCAACAAAGGGTAATATCCGGAAACCTCCTCGGATTCCATTGCCCAGCTATCTGTCACTTTATTGTGAAGATAGTGGAAAAGGAAGGTGGCTCCTACAAATGCCATCATTGCGATAAAGGAAAGGCCATCGTTGAAGATGCCTCTGCCGACAGTGGTCCCAAAGATGGACCCCCACCCACGAGGAGCATCGTGGAAAAAGAAGACGTTCCAACCACGTCTTCAAAGCAAGTGGATTGATGTGATATCTCCACTGACGTAAGGGATGACGCACAATCCCACTATCCTTCGCAAGACCCTTCCTCTATATAAGGAAGTTCATTTCATTTGGAGAGAACACGGGGGACTCTTGACCATGGTAGATCTGAGGGTAAATTTCTAGTTTTTCTCCTTCATTTTCTTGGTTAGGACCCTTTTCTCTTTTTATTTTTTTGAGCTTTGATCTTTCTTTAAACTGATCTATTTTTTAATTGATTGGTTATGGTGTAAATATTACATAGCTTTAACTGATAATCTGATTACTTTATTTCGTGTGTCTATGATGATGATGATAGTTACAGAACCGACGAACTAGTCTGTACCCGATCAACACCGAaACCCGTGGCGTCTTCGACCTCAATGGCGTCTGGAACTTCAAGCTGGACTACGGGAAAGGACTGGAAGAGAAGTGGTACGAAAGCAAGCTGACCGACACTATTAGTATGGCCGTCCCAAGCAGTTACAATGACATTGGCGTGACCAAGGAAATCCGCAACCATATCGGATATGTCTGGTACGAACGTGAGTTCACGGTGCCGGCCTATCTGAAGGATCAGCGTATCGTGCTCCGCTTCGGCTCTGCAACTCACAAAGCAATTGTCTATGTCAATGGTGAGCTGGTCGTGGAGCACAAGGGCGGATTCCTGCCATTCGAAGCGGAAATCAACAACTCGCTGCGTGATGGCATGAATCGCGTCACCGTCGCCGTGGACAACATCCTCGACGATAGCACCCTCCCGGTGGGGCTGTACAGCGAGCGCCACGAAGAGGGCCTCGGAAAAGTCATTCGTAACAAGCCGAACTTCGACTTCTTCAACTATGCAGGCCTGCACCGTCCGGTGAAAATCTACACGACCCCGTTTACGTACGTCGAGGACATCTCGGTTGTGACCGACTTCAATGGCCCAACCGGGACTGTGACCTATACGGTGGACTTTCAAGGCAAAGCCGAGACtGTGAAAGTGTCGGTCGTGGATGAGGAAGGCAAAGTGGTCGCAAGCACCGAGGGCCTGAGCGGTAACGTGGAGATTCCGAATGTCATCCTCTGGGAACCACTGAACACGTATCTCTACCAGATCAAAGTGGAACTGGTGAACGACGGACTGACCATCGATGTCTATGAAGAGCCGTTCGGCGTGCGGACCGTGGAAGTCAACGACGGCAAGTTCCTCATCAACAACAAACCGTTCTACTTCAAGGGCTTTGGCAAACATGAGGACACTCCTATCAACGGCCGTGGCTTTAACGAAGCGAGCAATGTGATGGATTTCAATATCCTCAAATGGATCGGCGCCAACAGCTTCCGGACCGCACACTATCCGTACTCTGAAGAGTTGATGCGTCTTGCGGATCGCGAGGGTCTGGTCGTGATCGACGAGACTCCGGCAGTTGGCGTGCACCTCAACTTCATGGCCACCACGGGACTCGGCGAAGGCAGCGAGCGCGTCAGTACCTGGGAGAAGATTCGGACGTTTGAGCACCATCAAGACGTTCTCCGTGAACTGGTGTCTCGTGACAAGAACCATCCAAGCGTCGTGATGTGGAGCATCGCCAACGAGGCGGCGACTGAGGAAGAGGGCGCGTACGAGTACTTCAAGCCGTTGGTGGAGCTGACCAAGGAACTCGACCCACAGAAGCGTCCGGTCACGATCGTGCTGTTTGTGATGGCTACCCCGGAGACGGACAAAGTCGCCGAACTGATTGACGTCATCGCGCTCAATCGCTATAACGGATGGTACTTCGATGGCGGTGATCTCGAAGCGGCCAAAGTCCATCTCCGCCAGGAATTTCACGCGTGGAACAAGCGTTGCCCAGGAAAGCCGATCATGATCACTGAGTACGGCGCAGACACCGTTGCGGGCTTTCACGACATTGATCCAGTGATGTTCACCGAGGAATATCAAGTCGAGTACTACCAGGCGAACCACGTCGTGTTCGATGAGTTTGAGAACTTCGTGGGTGAGCAAGCGTGGAACTTCGCGGACTTCGCGACCTCTCAGGGCGTGATGCGCGTCCAAGGAAACAAGAAGGGCGTGTTCACTCGTGACCGCAAGCCGAAGCTCGCCGCGCACGTCTTTCGCGAGCGCTGGACCAACATTCCAGATTTCGGCTACAAGAACGCTAGCCATCACCATCACCATCACGTGTGAATTGGTGACCAGCTCGAATTTCCCCGATCGTTCAAACATTTGGCAATAAAGTTTCTTAAGATTGAATCCTGTTGCCGGTCTTGCGATGATTATCATATAATTTCTGTTGAATTACGTTAAGCATGTAATAATTAACATGTAATGCATGACGTTATTTATGAGATGGGTTTTTATGATTAGAGTCCCGCAATTATACATTTAATACGCGATAGAAAACAAAATATAGCGCGCAAACTAGGATAAATTATCGCGCGCGGTGTCATCTATGTTACTAGATCGGGAATTAAACTATCGCGATCGCtgcggccgatcttagccagacgagcgggttcggcccattcggaccgcaaggaatcggtcaatacactacatggcgtgatttcatatgcgcgattgctgatccccatgtgtatcactggcaaactgtgatggacgacaccgtcagtgcgtccgtcgcgcaggctctcgatgagctgatgctttgggccgaggactgccccgaagtccggcacctcgtgcacgcggatttcggctccaacaatgtcctgacggacaatggccgcataacagcggtcattgactggagcgaggcgatgttcggggattcccaatacgaggtcgccaacatcttcttctggaggccgtggttggcttgtatggagcagcagacgcgctacttcgagcggaggcatccggagcttgcaggatcgccacgactccgggcgtatatgctccgcattggtcttgaccaactctatcagagcttggttgacggcaatttcgatgatgcagcttgggcgcagggtcgatgcgacgcaatcgtccgatccggagccgggactgtcgggcgtacacaaatcgcccgcagaagcgcggccgtctggaccgatggctgtgtagaagtactcgccgatagtggaaaccgacgccccagcactcgtccgagggcaaagaaatagagtagatgccgaccggatctgtcgatcgacaagctcgagtttctccataataatgtgtgagtagttcccagataagggaattagggttcctatagggtttcgctcatgtgttgagcatataagaaacccttagtatgtatttgtatttgtaaaatacttctatcaataaaatttctaattcctaaaaccaaaatccagtactaaaatccagatcccccgaattaattcggcgttaattcagtacattaaaaacgtccgcaatgtgttattaagttgtctaagcgtcaatttgtttacaccacaatatatcctgccaccagccagccaacagctccccgaccggcagctcggcacaaaatcaccactcgatacaggcagcccatcagtccgggacggcgtcagcgggagagccgttgtaaggcggcagactttgctcatgttaccgatgctattcggaagaacggcaactaagctgccgggtttgaaacacggatgatctcgcggagggtagcatgttgattgtaacgatgacagagcgttgctgcctgtgatcaccgcggtttcaaaatcggctccgtcgatactatgttatacgccaactttgaaaacaactttgaaaaagctgttttctggtatttaaggttttagaatgcaaggaacagtgaattggagttcgtcttgttataattagcttcttggggtatctttaaatactgtagaaaagaggaaggaaataataaatggctaaaatgagaatatcaccggaattgaaaaaactgatcgaaaaataccgctgcgtaaaagatacggaaggaatgtctcctgctaaggtatataagctggtgggagaaaatgaaaacctatatttaaaaatgacggacagccggtataaagggaccacctatgatgtggaacgggaaaaggacatgatgctatggctggaaggaaagctgcctgttccaaaggtcctgcactttgaacggcatgatggctggagcaatctgctcatgagtgaggccgatggcgtcctttgctcggaagagtatgaagatgaacaaagccctgaaaagattatcgagctgtatgcggagtgcatcaggctctttcactccatcgacatatcggattgtccctatacgaatagcttagacagccgcttagccgaattggattacttactgaataacgatctggccgatgtggattgcgaaaactgggaagaagacactccatttaaagatccgcgcgagctgtatgattttttaaagacggaaaagcccgaagaggaacttgtcttttcccacggcgacctgggagacagcaacatctttgtgaaagatggcaaagtaagtggctttattgatcttgggagaagcggcagggcggacaagtggtatgacattgccttctgcgtccggtcgatcagggaggatatcggggaagaacagtatgtcgagctattttttgacttactggggatcaagcctgattgggagaaaataaaatattatattttactggatgaattgttttagtacctagaatgcatgaccaaaatcccttaacgtgagttttcgttccactgagcgtcagaccccgtagaaaagatcaaaggatcttcttgagatcctttttttctgcgcgtaatctgctgcttgcaaacaaaaaaaccaccgctaccagcggtggtttgtttgccggatcaagagctaccaactctttttccgaaggtaactggcttcagcagagcgcagataccaaatactgtccttctagtgtagccgtagttaggccaccacttcaagaactctgtagcaccgcctacatacctcgctctgctaatcctgttaccagtggctgctgccagtggcgataagtcgtgtcttaccgggttggactcaagacgatagttaccggataaggcgcagcggtcgggctgaacggggggttcgtgcacacagcccagcttggagcgaacgacctacaccgaactgagatacctacagcgtgagctatgagaaagcgccacgcttcccgaagggagaaaggcggacaggtatccggtaagcggcagggtcggaacaggagagcgcacgagggagcttccagggggaaacgcctggtatctttatagtcctgtcgggtttcgccacctctgacttgagcgtcgatttttgtgatgctcgtcaggggggcggagcctatggaaaaacgccagcaacgcggcctttttacggttcctggccttttgctggccttttgctcacatgttctttcctgcgttatcccctgattctgtggataaccgtattaccgcctttgagtgagctgataccgctcgccgcagccgaacgaccgagcgcagcgagtcagtgagcgaggaagcggaagagcgcctgatgcggtattttctccttacgcatctgtgcggtatttcacaccgcatatggtgcactctcagtacaatctgctctgatgccgcatagttaagccagtatacactccgctatcgctacgtgactgggtcatggctgcgccccgacacccgccaacacccgctgacgcgccctgacgggcttgtctgctcccggcatccgcttacagacaagctgtgaccgtctccgggagctgcatgtgtcagaggttttcaccgtcatcaccgaaacgcgcgaggcagggtgccttgatgtgggcgccggcggtcgagtggcgacggcgcggcttgtccgcgccctggtagattgcctggccgtaggccagccatttttgagcggccagcggccgcgataggccgacgcgaagcggcggggcgtagggagcgcagcgaccgaagggtaggcgctttttgcagctcttcggctgtgcgctggccagacagttatgcacaggccaggcgggttttaagagttttaataagttttaaagagttttaggcggaaaaatcgccttttttctcttttatatcagtcacttacatgtgtgaccggttcccaatgtacggctttgggttcccaatgtacgggttccggttcccaatgtacggctttgggttcccaatgtacgtgctatccacaggaaacagaccttttcgacctttttcccctgctagggcaatttgccctagcatctgctccgtacattaggaaccggcggatgcttcgccctcgatcaggttgcggtagcgcatgactaggatcgggccagcctgccccgcctcctccttcaaatcgtactccggcaggtcatttgacccgatcagcttgcgcacggtgaaacagaacttcttgaactctccggcgctgccactgcgttcgtagatcgtcttgaacaaccatctggcttctgccttgcctgcggcgcggcgtgccaggcggtagagaaaacggccgatgccgggatcgatcaaaaagtaatcggggtgaaccgtcagcacgtccgggttcttgccttctgtgatctcgcggtacatccaatcagctagctcgatctcgatgtactccggccgcccggtttcgctctttacgatcttgtagcggctaatcaaggcttcaccctcggataccgtcaccaggcggccgttcttggccttcttcgtacgctgcatggcaacgtgcgtggtgtttaaccgaatgcaggtttctaccaggtcgtctttctgctttccgccatcggctcgccggcagaacttgagtacgtccgcaacgtgtggacggaacacgcggccgggcttgtctcccttcccttcccggtatcggttcatggattcggttagatgggaaaccgccatcagtaccaggtcgtaatcccacacactggccatgccggccggccctgcggaaacctctacgtgcccgtctggaagctcgtagcggatcacctcgccagctcgtcggtcacgcttcgacagacggaaaacggccacgtccatgatgctgcgactatcgcgggtgcccacgtcatagagcatcggaacgaaaaaatctggttgctcgtcgcccttgggcggcttcctaatcgacggcgcaccggctgccggcggttgccgggattctttgcggattcgatcagcggccgcttgccacgattcaccggggcgtgcttctgcctcgatgcgttgccgctgggcggcctgcgcggccttcaacttctccaccaggtcatcacccagcgccgcgccgatttgtaccgggccggatggtttgcgaccgctcacgccgattcctcgggcttgggggttccagtgccattgcagggccggcagacaacccagccgcttacgcctggccaaccgcccgttcctccacacatggggcattccacggcgtcggtgcctggttgttcttgattttccatgccgcctcctttagccgctaaaattcatctactcatttattcatttgctcatttactctggtagctgcgcgatgtattcagatagcagctcggtaatggtcttgccttggcgtaccgcgtacatcttcagcttggtgtgatcctccgccggcaactgaaagttgacccgcttcatggctggcgtgtctgccaggctggccaacgttgcagccttgctgctgcgtgcgctcggacggccggcacttagcgtgtttgtgcttttgctcattttctctttacctcattaactcaaatgagttttgatttaatttcagcggccagcgcctggacctcgcgggcagcgtcgccctcgggttctgattcaagaacggttgtgccggcggcggcagtgcctgggtagctcacgcgctgcgtgatacgggactcaagaatgggcagctcgtacccggccagcgcctcggcaacctcaccgccgatgcgcgtgcctttgatcgcccgcgacacgacaaaggccgcttgtagccttccatccgtgacctcaatgcgctgcttaaccagctccaccaggtcggcggtggcccatatgtcgtaagggcttggctgcaccggaatcagcacgaagtcggctgccttgatcgcggacacagccaagtccgccgcctggggcgctccgtcgatcactacgaagtcgcgccggccgatggccttcacgtcgcggtcaatcgtcgggcggtcgatgccgacaacggttagcggttgatcttcccgcacggccgcccaatcgcgggcactgccctggggatcggaatcgactaacagaacatcggccccggcgagttgcagggcgcgggctagatgggttgcgatggtcgtcttgcctgacccgcctttctggttaagtacagcgataaccttcatgcgttccccttgcgtatttgtttatttactcatcgcatcatatacgcagcgaccgcatgacgcaagctgttttactcaaatacacatcacctttttagacggcggcgctcggtttcttcagcggccaagctggccggccaggccgccagcttggcatcagacaaaccggccaggatttcatgcagccgcacggttgagacgtgcgcgggcggctcgaacacgtacccggccgcgatcatctccgcctcgatctcttcggtaatgaaaaacggttcgtcctggccgtcctggtgcggtttcatgcttgttcctcttggcgttcattctcggcggccgccagggcgtcggcctcggtcaatgcgtcctcacggaaggcaccgcgccgcctggcctcggtgggcgtcacttcctcgctgcgctcaagtgcgcggtacagggtcgagcgatgcacgccaagcagtgcagccgcctctttcacggtgcggccttcctggtcgatcagctcgcgggcgtgcgcgatctgtgccggggtgagggtagggcgggggccaaacttcacgcctcgggccttggcggcctcgcgcccgctccgggtgcggtcgatgattagggaacgctcgaactcggcaatgccggcgaacacggtcaacaccatgcggccggccggcgtggtggtgtcggcccacggctctgccaggctacgcaggcccgcgccggcctcctggatgcgctcggcaatgtccagtaggtcgcgggtgctgcgggccaggcggtctagcctggtcactgtcacaacgtcgccagggcgtaggtggtcaagcatcctggccagctccgggcggtcgcgcctggtgccggtgatcttctcggaaaacagcttggtgcagccggccgcgtgcagttcggcccgttggttggtcaagtcctggtcgtcggtgctgacgcgggcatagcccagcaggccagcggcggcgctcttgttcatggcgtaatgtctccggttctagtcgcaagtattctactttatgcgactaaaacacgcgacaagaaaacgccaggaaaagggcagggcggcagcctgtcgcgtaacttaggacttgtgcgacatgtcgttttcagaagacggctgcactgaacgtcagaagccgactgcactatagcagcggaggggttggatcaaagtactttgatcccgaggggaaccctgtggttggcatgcacatacaaatggacgaacggataaaccttttcacgcccttttaaatatccgttattctaa

>AF354045.1 Binary vector pCAMBIA-1305.1, complete sequence, 11846bp

CATGGTAGATCTGAGGGTAAATTTCTAGTTTTTCTCCTTCATTTTCTTGGTTAGGACCCTTTTCTCTTTTTATTTTTTTGAGCTTTGATCTTTCTTTAAACTGATCTATTTTTTAATTGATTGGTTATGGTGTAAATATTACATAGCTTTAACTGATAATCTGATTACTTTATTTCGTGTGTCTATGATGATGATGATAGTTACAGAACCGACGAACTAGTCTGTACCCGATCAACACCGAGACCCGTGGCGTCTTCGACCTCAATGGCGTCTGGAACTTCAAGCTGGACTACGGGAAAGGACTGGAAGAGAAGTGGTACGAAAGCAAGCTGACCGACACTATTAGTATGGCCGTCCCAAGCAGTTACAATGACATTGGCGTGACCAAGGAAATCCGCAACCATATCGGATATGTCTGGTACGAACGTGAGTTCACGGTGCCGGCCTATCTGAAGGATCAGCGTATCGTGCTCCGCTTCGGCTCTGCAACTCACAAAGCAATTGTCTATGTCAATGGTGAGCTGGTCGTGGAGCACAAGGGCGGATTCCTGCCATTCGAAGCGGAAATCAACAACTCGCTGCGTGATGGCATGAATCGCGTCACCGTCGCCGTGGACAACATCCTCGACGATAGCACCCTCCCGGTGGGGCTGTACAGCGAGCGCCACGAAGAGGGCCTCGGAAAAGTCATTCGTAACAAGCCGAACTTCGACTTCTTCAACTATGCAGGCCTGCACCGTCCGGTGAAAATCTACACGACCCCGTTTACGTACGTCGAGGACATCTCGGTTGTGACCGACTTCAATGGCCCAACCGGGACTGTGACCTATACGGTGGACTTTCAAGGCAAAGCCGAGACCGTGAAAGTGTCGGTCGTGGATGAGGAAGGCAAAGTGGTCGCAAGCACCGAGGGCCTGAGCGGTAACGTGGAGATTCCGAATGTCATCCTCTGGGAACCACTGAACACGTATCTCTACCAGATCAAAGTGGAACTGGTGAACGACGGACTGACCATCGATGTCTATGAAGAGCCGTTCGGCGTGCGGACCGTGGAAGTCAACGACGGCAAGTTCCTCATCAACAACAAACCGTTCTACTTCAAGGGCTTTGGCAAACATGAGGACACTCCTATCAACGGCCGTGGCTTTAACGAAGCGAGCAATGTGATGGATTTCAATATCCTCAAATGGATCGGCGCCAACAGCTTCCGGACCGCACACTATCCGTACTCTGAAGAGTTGATGCGTCTTGCGGATCGCGAGGGTCTGGTCGTGATCGACGAGACTCCGGCAGTTGGCGTGCACCTCAACTTCATGGCCACCACGGGACTCGGCGAAGGCAGCGAGCGCGTCAGTACCTGGGAGAAGATTCGGACGTTTGAGCACCATCAAGACGTTCTCCGTGAACTGGTGTCTCGTGACAAGAACCATCCAAGCGTCGTGATGTGGAGCATCGCCAACGAGGCGGCGACTGAGGAAGAGGGCGCGTACGAGTACTTCAAGCCGTTGGTGGAGCTGACCAAGGAACTCGACCCACAGAAGCGTCCGGTCACGATCGTGCTGTTTGTGATGGCTACCCCGGAGACGGACAAAGTCGCCGAACTGATTGACGTCATCGCGCTCAATCGCTATAACGGATGGTACTTCGATGGCGGTGATCTCGAAGCGGCCAAAGTCCATCTCCGCCAGGAATTTCACGCGTGGAACAAGCGTTGCCCAGGAAAGCCGATCATGATCACTGAGTACGGCGCAGACACCGTTGCGGGCTTTCACGACATTGATCCAGTGATGTTCACCGAGGAATATCAAGTCGAGTACTACCAGGCGAACCACGTCGTGTTCGATGAGTTTGAGAACTTCGTGGGTGAGCAAGCGTGGAACTTCGCGGACTTCGCGACCTCTCAGGGCGTGATGCGCGTCCAAGGAAACAAGAAGGGCGTGTTCACTCGTGACCGCAAGCCGAAGCTCGCCGCGCACGTCTTTCGCGAGCGCTGGACCAACATTCCAGATTTCGGCTACAAGAACGCTAGCCATCACCATCACCATCACGTGTGAATTGGTGACCAGCTCGAATTTCCCCGATCGTTCAAACATTTGGCAATAAAGTTTCTTAAGATTGAATCCTGTTGCCGGTCTTGCGATGATTATCATATAATTTCTGTTGAATTACGTTAAGCATGTAATAATTAACATGTAATGCATGACGTTATTTATGAGATGGGTTTTTATGATTAGAGTCCCGCAATTATACATTTAATACGCGATAGAAAACAAAATATAGCGCGCAAACTAGGATAAATTATCGCGCGCGGTGTCATCTATGTTACTAGATCGGGAATTAAACTATCAGTGTTTGACAGGATATATTGGCGGGTAAACCTAAGAGAAAAGAGCGTTTATTAGAATAACGGATATTTAAAAGGGCGTGAAAAGGTTTATCCGTTCGTCCATTTGTATGTGCATGCCAACCACAGGGTTCCCCTCGGGATCAAAGTACTTTGATCCAACCCCTCCGCTGCTATAGTGCAGTCGGCTTCTGACGTTCAGTGCAGCCGTCTTCTGAAAACGACATGTCGCACAAGTCCTAAGTTACGCGACAGGCTGCCGCCCTGCCCTTTTCCTGGCGTTTTCTTGTCGCGTGTTTTAGTCGCATAAAGTAGAATACTTGCGACTAGAACCGGAGACATTACGCCATGAACAAGAGCGCCGCCGCTGGCCTGCTGGGCTATGCCCGCGTCAGCACCGACGACCAGGACTTGACCAACCAACGGGCCGAACTGCACGCGGCCGGCTGCACCAAGCTGTTTTCCGAGAAGATCACCGGCACCAGGCGCGACCGCCCGGAGCTGGCCAGGATGCTTGACCACCTACGCCCTGGCGACGTTGTGACAGTGACCAGGCTAGACCGCCTGGCCCGCAGCACCCGCGACCTACTGGACATTGCCGAGCGCATCCAGGAGGCCGGCGCGGGCCTGCGTAGCCTGGCAGAGCCGTGGGCCGACACCACCACGCCGGCCGGCCGCATGGTGTTGACCGTGTTCGCCGGCATTGCCGAGTTCGAGCGTTCCCTAATCATCGACCGCACCCGGAGCGGGCGCGAGGCCGCCAAGGCCCGAGGCGTGAAGTTTGGCCCCCGCCCTACCCTCACCCCGGCACAGATCGCGCACGCCCGCGAGCTGATCGACCAGGAAGGCCGCACCGTGAAAGAGGCGGCTGCACTGCTTGGCGTGCATCGCTCGACCCTGTACCGCGCACTTGAGCGCAGCGAGGAAGTGACGCCCACCGAGGCCAGGCGGCGCGGTGCCTTCCGTGAGGACGCATTGACCGAGGCCGACGCCCTGGCGGCCGCCGAGAATGAACGCCAAGAGGAACAAGCATGAAACCGCACCAGGACGGCCAGGACGAACCGTTTTTCATTACCGAAGAGATCGAGGCGGAGATGATCGCGGCCGGGTACGTGTTCGAGCCGCCCGCGCACGTCTCAACCGTGCGGCTGCATGAAATCCTGGCCGGTTTGTCTGATGCCAAGCTGGCGGCCTGGCCGGCCAGCTTGGCCGCTGAAGAAACCGAGCGCCGCCGTCTAAAAAGGTGATGTGTATTTGAGTAAAACAGCTTGCGTCATGCGGTCGCTGCGTATATGATGCGATGAGTAAATAAACAAATACGCAAGGGGAACGCATGAAGGTTATCGCTGTACTTAACCAGAAAGGCGGGTCAGGCAAGACGACCATCGCAACCCATCTAGCCCGCGCCCTGCAACTCGCCGGGGCCGATGTTCTGTTAGTCGATTCCGATCCCCAGGGCAGTGCCCGCGATTGGGCGGCCGTGCGGGAAGATCAACCGCTAACCGTTGTCGGCATCGACCGCCCGACGATTGACCGCGACGTGAAGGCCATCGGCCGGCGCGACTTCGTAGTGATCGACGGAGCGCCCCAGGCGGCGGACTTGGCTGTGTCCGCGATCAAGGCAGCCGACTTCGTGCTGATTCCGGTGCAGCCAAGCCCTTACGACATATGGGCCACCGCCGACCTGGTGGAGCTGGTTAAGCAGCGCATTGAGGTCACGGATGGAAGGCTACAAGCGGCCTTTGTCGTGTCGCGGGCGATCAAAGGCACGCGCATCGGCGGTGAGGTTGCCGAGGCGCTGGCCGGGTACGAGCTGCCCATTCTTGAGTCCCGTATCACGCAGCGCGTGAGCTACCCAGGCACTGCCGCCGCCGGCACAACCGTTCTTGAATCAGAACCCGAGGGCGACGCTGCCCGCGAGGTCCAGGCGCTGGCCGCTGAAATTAAATCAAAACTCATTTGAGTTAATGAGGTAAAGAGAAAATGAGCAAAAGCACAAACACGCTAAGTGCCGGCCGTCCGAGCGCACGCAGCAGCAAGGCTGCAACGTTGGCCAGCCTGGCAGACACGCCAGCCATGAAGCGGGTCAACTTTCAGTTGCCGGCGGAGGATCACACCAAGCTGAAGATGTACGCGGTACGCCAAGGCAAGACCATTACCGAGCTGCTATCTGAATACATCGCGCAGCTACCAGAGTAAATGAGCAAATGAATAAATGAGTAGATGAATTTTAGCGGCTAAAGGAGGCGGCATGGAAAATCAAGAACAACCAGGCACCGACGCCGTGGAATGCCCCATGTGTGGAGGAACGGGCGGTTGGCCAGGCGTAAGCGGCTGGGTTGTCTGCCGGCCCTGCAATGGCACTGGAACCCCCAAGCCCGAGGAATCGGCGTGACGGTCGCAAACCATCCGGCCCGGTACAAATCGGCGCGGCGCTGGGTGATGACCTGGTGGAGAAGTTGAAGGCCGCGCAGGCCGCCCAGCGGCAACGCATCGAGGCAGAAGCACGCCCCGGTGAATCGTGGCAAGCGGCCGCTGATCGAATCCGCAAAGAATCCCGGCAACCGCCGGCAGCCGGTGCGCCGTCGATTAGGAAGCCGCCCAAGGGCGACGAGCAACCAGATTTTTTCGTTCCGATGCTCTATGACGTGGGCACCCGCGATAGTCGCAGCATCATGGACGTGGCCGTTTTCCGTCTGTCGAAGCGTGACCGACGAGCTGGCGAGGTGATCCGCTACGAGCTTCCAGACGGGCACGTAGAGGTTTCCGCAGGGCCGGCCGGCATGGCCAGTGTGTGGGATTACGACCTGGTACTGATGGCGGTTTCCCATCTAACCGAATCCATGAACCGATACCGGGAAGGGAAGGGAGACAAGCCCGGCCGCGTGTTCCGTCCACACGTTGCGGACGTACTCAAGTTCTGCCGGCGAGCCGATGGCGGAAAGCAGAAAGACGACCTGGTAGAAACCTGCATTCGGTTAAACACCACGCACGTTGCCATGCAGCGTACGAAGAAGGCCAAGAACGGCCGCCTGGTGACGGTATCCGAGGGTGAAGCCTTGATTAGCCGCTACAAGATCGTAAAGAGCGAAACCGGGCGGCCGGAGTACATCGAGATCGAGCTAGCTGATTGGATGTACCGCGAGATCACAGAAGGCAAGAACCCGGACGTGCTGACGGTTCACCCCGATTACTTTTTGATCGATCCCGGCATCGGCCGTTTTCTCTACCGCCTGGCACGCCGCGCCGCAGGCAAGGCAGAAGCCAGATGGTTGTTCAAGACGATCTACGAACGCAGTGGCAGCGCCGGAGAGTTCAAGAAGTTCTGTTTCACCGTGCGCAAGCTGATCGGGTCAAATGACCTGCCGGAGTACGATTTGAAGGAGGAGGCGGGGCAGGCTGGCCCGATCCTAGTCATGCGCTACCGCAACCTGATCGAGGGCGAAGCATCCGCCGGTTCCTAATGTACGGAGCAGATGCTAGGGCAAATTGCCCTAGCAGGGGAAAAAGGTCGAAAAGGTCTCTTTCCTGTGGATAGCACGTACATTGGGAACCCAAAGCCGTACATTGGGAACCGGAACCCGTACATTGGGAACCCAAAGCCGTACATTGGGAACCGGTCACACATGTAAGTGACTGATATAAAAGAGAAAAAAGGCGATTTTTCCGCCTAAAACTCTTTAAAACTTATTAAAACTCTTAAAACCCGCCTGGCCTGTGCATAACTGTCTGGCCAGCGCACAGCCGAAGAGCTGCAAAAAGCGCCTACCCTTCGGTCGCTGCGCTCCCTACGCCCCGCCGCTTCGCGTCGGCCTATCGCGGCCGCTGGCCGCTCAAAAATGGCTGGCCTACGGCCAGGCAATCTACCAGGGCGCGGACAAGCCGCGCCGTCGCCACTCGACCGCCGGCGCCCACATCAAGGCACCCTGCCTCGCGCGTTTCGGTGATGACGGTGAAAACCTCTGACACATGCAGCTCCCGGAGACGGTCACAGCTTGTCTGTAAGCGGATGCCGGGAGCAGACAAGCCCGTCAGGGCGCGTCAGCGGGTGTTGGCGGGTGTCGGGGCGCAGCCATGACCCAGTCACGTAGCGATAGCGGAGTGTATACTGGCTTAACTATGCGGCATCAGAGCAGATTGTACTGAGAGTGCACCATATGCGGTGTGAAATACCGCACAGATGCGTAAGGAGAAAATACCGCATCAGGCGCTCTTCCGCTTCCTCGCTCACTGACTCGCTGCGCTCGGTCGTTCGGCTGCGGCGAGCGGTATCAGCTCACTCAAAGGCGGTAATACGGTTATCCACAGAATCAGGGGATAACGCAGGAAAGAACATGTGAGCAAAAGGCCAGCAAAAGGCCAGGAACCGTAAAAAGGCCGCGTTGCTGGCGTTTTTCCATAGGCTCCGCCCCCCTGACGAGCATCACAAAAATCGACGCTCAAGTCAGAGGTGGCGAAACCCGACAGGACTATAAAGATACCAGGCGTTTCCCCCTGGAAGCTCCCTCGTGCGCTCTCCTGTTCCGACCCTGCCGCTTACCGGATACCTGTCCGCCTTTCTCCCTTCGGGAAGCGTGGCGCTTTCTCATAGCTCACGCTGTAGGTATCTCAGTTCGGTGTAGGTCGTTCGCTCCAAGCTGGGCTGTGTGCACGAACCCCCCGTTCAGCCCGACCGCTGCGCCTTATCCGGTAACTATCGTCTTGAGTCCAACCCGGTAAGACACGACTTATCGCCACTGGCAGCAGCCACTGGTAACAGGATTAGCAGAGCGAGGTATGTAGGCGGTGCTACAGAGTTCTTGAAGTGGTGGCCTAACTACGGCTACACTAGAAGGACAGTATTTGGTATCTGCGCTCTGCTGAAGCCAGTTACCTTCGGAAAAAGAGTTGGTAGCTCTTGATCCGGCAAACAAACCACCGCTGGTAGCGGTGGTTTTTTTGTTTGCAAGCAGCAGATTACGCGCAGAAAAAAAGGATCTCAAGAAGATCCTTTGATCTTTTCTACGGGGTCTGACGCTCAGTGGAACGAAAACTCACGTTAAGGGATTTTGGTCATGCATTCTAGGTACTAAAACAATTCATCCAGTAAAATATAATATTTTATTTTCTCCCAATCAGGCTTGATCCCCAGTAAGTCAAAAAATAGCTCGACATACTGTTCTTCCCCGATATCCTCCCTGATCGACCGGACGCAGAAGGCAATGTCATACCACTTGTCCGCCCTGCCGCTTCTCCCAAGATCAATAAAGCCACTTACTTTGCCATCTTTCACAAAGATGTTGCTGTCTCCCAGGTCGCCGTGGGAAAAGACAAGTTCCTCTTCGGGCTTTTCCGTCTTTAAAAAATCATACAGCTCGCGCGGATCTTTAAATGGAGTGTCTTCTTCCCAGTTTTCGCAATCCACATCGGCCAGATCGTTATTCAGTAAGTAATCCAATTCGGCTAAGCGGCTGTCTAAGCTATTCGTATAGGGACAATCCGATATGTCGATGGAGTGAAAGAGCCTGATGCACTCCGCATACAGCTCGATAATCTTTTCAGGGCTTTGTTCATCTTCATACTCTTCCGAGCAAAGGACGCCATCGGCCTCACTCATGAGCAGATTGCTCCAGCCATCATGCCGTTCAAAGTGCAGGACCTTTGGAACAGGCAGCTTTCCTTCCAGCCATAGCATCATGTCCTTTTCCCGTTCCACATCATAGGTGGTCCCTTTATACCGGCTGTCCGTCATTTTTAAATATAGGTTTTCATTTTCTCCCACCAGCTTATATACCTTAGCAGGAGACATTCCTTCCGTATCTTTTACGCAGCGGTATTTTTCGATCAGTTTTTTCAATTCCGGTGATATTCTCATTTTAGCCATTTATTATTTCCTTCCTCTTTTCTACAGTATTTAAAGATACCCCAAGAAGCTAATTATAACAAGACGAACTCCAATTCACTGTTCCTTGCATTCTAAAACCTTAAATACCAGAAAACAGCTTTTTCAAAGTTGTTTTCAAAGTTGGCGTATAACATAGTATCGACGGAGCCGATTTTGAAACCGCGGTGATCACAGGCAGCAACGCTCTGTCATCGTTACAATCAACATGCTACCCTCCGCGAGATCATCCGTGTTTCAAACCCGGCAGCTTAGTTGCCGTTCTTCCGAATAGCATCGGTAACATGAGCAAAGTCTGCCGCCTTACAACGGCTCTCCCGCTGACGCCGTCCCGGACTGATGGGCTGCCTGTATCGAGTGGTGATTTTGTGCCGAGCTGCCGGTCGGGGAGCTGTTGGCTGGCTGGTGGCAGGATATATTGTGGTGTAAACAAATTGACGCTTAGACAACTTAATAACACATTGCGGACGTTTTTAATGTACTGAATTAACGCCGAATTAATTCGGGGGATCTGGATTTTAGTACTGGATTTTGGTTTTAGGAATTAGAAATTTTATTGATAGAAGTATTTTACAAATACAAATACATACTAAGGGTTTCTTATATGCTCAACACATGAGCGAAACCCTATAGGAACCCTAATTCCCTTATCTGGGAACTACTCACACATTATTATGGAGAAACTCGAGCTTGTCGATCGACAGATCCGGTCGGCATCTACTCTATTTCTTTGCCCTCGGACGAGTGCTGGGGCGTCGGTTTCCACTATCGGCGAGTACTTCTACACAGCCATCGGTCCAGACGGCCGCGCTTCTGCGGGCGATTTGTGTACGCCCGACAGTCCCGGCTCCGGATCGGACGATTGCGTCGCATCGACCCTGCGCCCAAGCTGCATCATCGAAATTGCCGTCAACCAAGCTCTGATAGAGTTGGTCAAGACCAATGCGGAGCATATACGCCCGGAGTCGTGGCGATCCTGCAAGCTCCGGATGCCTCCGCTCGAAGTAGCGCGTCTGCTGCTCCATACAAGCCAACCACGGCCTCCAGAAGAAGATGTTGGCGACCTCGTATTGGGAATCCCCGAACATCGCCTCGCTCCAGTCAATGACCGCTGTTATGCGGCCATTGTCCGTCAGGACATTGTTGGAGCCGAAATCCGCGTGCACGAGGTGCCGGACTTCGGGGCAGTCCTCGGCCCAAAGCATCAGCTCATCGAGAGCCTGCGCGACGGACGCACTGACGGTGTCGTCCATCACAGTTTGCCAGTGATACACATGGGGATCAGCAATCGCGCATATGAAATCACGCCATGTAGTGTATTGACCGATTCCTTGCGGTCCGAATGGGCCGAACCCGCTCGTCTGGCTAAGATCGGCCGCAGCGATCGCATCCATAGCCTCCGCGACCGGTTGTAGAACAGCGGGCAGTTCGGTTTCAGGCAGGTCTTGCAACGTGACACCCTGTGCACGGCGGGAGATGCAATAGGTCAGGCTCTCGCTAAACTCCCCAATGTCAAGCACTTCCGGAATCGGGAGCGCGGCCGATGCAAAGTGCCGATAAACATAACGATCTTTGTAGAAACCATCGGCGCAGCTATTTACCCGCAGGACATATCCACGCCCTCCTACATCGAAGCTGAAAGCACGAGATTCTTCGCCCTCCGAGAGCTGCATCAGGTCGGAGACGCTGTCGAACTTTTCGATCAGAAACTTCTCGACAGACGTCGCGGTGAGTTCAGGCTTTTTCATATCTCATTGCCCCCCGGGATCTGCGAAAGCTCGAGAGAGATAGATTTGTAGAGAGAGACTGGTGATTTCAGCGTGTCCTCTCCAAATGAAATGAACTTCCTTATATAGAGGAAGGTCTTGCGAAGGATAGTGGGATTGTGCGTCATCCCTTACGTCAGTGGAGATATCACATCAATCCACTTGCTTTGAAGACGTGGTTGGAACGTCTTCTTTTTCCACGATGCTCCTCGTGGGTGGGGGTCCATCTTTGGGACCACTGTCGGCAGAGGCATCTTGAACGATAGCCTTTCCTTTATCGCAATGATGGCATTTGTAGGTGCCACCTTCCTTTTCTACTGTCCTTTTGATGAAGTGACAGATAGCTGGGCAATGGAATCCGAGGAGGTTTCCCGATATTACCCTTTGTTGAAAAGTCTCAATAGCCCTTTGGTCTTCTGAGACTGTATCTTTGATATTCTTGGAGTAGACGAGAGTGTCGTGCTCCACCATGTTATCACATCAATCCACTTGCTTTGAAGACGTGGTTGGAACGTCTTCTTTTTCCACGATGCTCCTCGTGGGTGGGGGTCCATCTTTGGGACCACTGTCGGCAGAGGCATCTTGAACGATAGCCTTTCCTTTATCGCAATGATGGCATTTGTAGGTGCCACCTTCCTTTTCTACTGTCCTTTTGATGAAGTGACAGATAGCTGGGCAATGGAATCCGAGGAGGTTTCCCGATATTACCCTTTGTTGAAAAGTCTCAATAGCCCTTTGGTCTTCTGAGACTGTATCTTTGATATTCTTGGAGTAGACGAGAGTGTCGTGCTCCACCATGTTGGCAAGCTGCTCTAGCCAATACGCAAACCGCCTCTCCCCGCGCGTTGGCCGATTCATTAATGCAGCTGGCACGACAGGTTTCCCGACTGGAAAGCGGGCAGTGAGCGCAACGCAATTAATGTGAGTTAGCTCACTCATTAGGCACCCCAGGCTTTACACTTTATGCTTCCGGCTCGTATGTTGTGTGGAATTGTGAGCGGATAACAATTTCACACAGGAAACAGCTATGACCATGATTACGAATTCGAGCTCGGTACCCGGGGATCCTCTAGAGTCGACCTGCAGGCATGCAAGCTTGGCACTGGCCGTCGTTTTACAACGTCGTGACTGGGAAAACCCTGGCGTTACCCAACTTAATCGCCTTGCAGCACATCCCCCTTTCGCCAGCTGGCGTAATAGCGAAGAGGCCCGCACCGATCGCCCTTCCCAACAGTTGCGCAGCCTGAATGGCGAATGCTAGAGCAGCTTGAGCTTGGATCAGATTGTCGTTTCCCGCCTTCAGTTTAGCTTCATGGAGTCAAAGATTCAAATAGAGGACCTAACAGAACTCGCCGTAAAGACTGGCGAACAGTTCATACAGAGTCTCTTACGACTCAATGACAAGAAGAAAATCTTCGTCAACATGGTGGAGCACGACACACTTGTCTACTCCAAAAATATCAAAGATACAGTCTCAGAAGACCAAAGGGCAATTGAGACTTTTCAACAAAGGGTAATATCCGGAAACCTCCTCGGATTCCATTGCCCAGCTATCTGTCACTTTATTGTGAAGATAGTGGAAAAGGAAGGTGGCTCCTACAAATGCCATCATTGCGATAAAGGAAAGGCCATCGTTGAAGATGCCTCTGCCGACAGTGGTCCCAAAGATGGACCCCCACCCACGAGGAGCATCGTGGAAAAAGAAGACGTTCCAACCACGTCTTCAAAGCAAGTGGATTGATGTGATATCTCCACTGACGTAAGGGATGACGCACAATCCCACTATCCTTCGCAAGACCCTTCCTCTATATAAGGAAGTTCATTTCATTTGGAGAGAACACGGGGGACTCTTGAC

>pYGUS1305, complete sequence, 11739bp

GGTACCTCTGAATCGAGCTTTCGGAATTTTAACATAGAGAAGTTAGAGAGAGAATGAAAAGCCAAAGGAGGCGAAAATCGAACAAGGAAGAAGAAAGACAACTTTCGACAAAGACTGGTCGGTCGGTTTTGGTAGACAATTGAAATTAGATGGATGGTCCGGTTCGGTATACTATAAGATTAAAAACAGTTTTAAATTCAGCTAAACCGAACTCATTTGATTTTATTAAACCGGAATCATCCGATTCGAGTTTGTAAAAAATACCGAAATTGAAAACACTAAACAAAAACTGTATTAAACTGTTACTGAAATAAGAGAATCTCCCAATTCGGTTTACGTACTACTCTTCAGAAATCAGAACCAAAAATTCAGAAATCGGATTGAACCAAACTTAAATTGACGGTCCGGTTAGTCTTCGGCTCTACAAATTAAAGGCCCAAGTTTCTGCTTTAAAAGAACGAAATAGTTAATGGGCTCAAACCATAGACCAGGTAAGTCATGGGCTTGGTTAGTCCGGGTCAACCCGGTAGACCCGATTCCTGAAGAAAACCTAGTGGAAGGTTTAAAGTTGTAAACTTTCCGACCAAATAAACAAAATCGTTTTCCAGCTTCTTCCGTCGCCACTAAACCCTGAGGCTAAACCTAGACGAGTCAAAGTGTAAAATCGTTAAACCCTAAGAGGGAGTGAGAGAGACCATGGTAGATCTGAGGGTAAATTTCTAGTTTTTCTCCTTCATTTTCTTGGTTAGGACCCTTTTCTCTTTTTATTTTTTTGAGCTTTGATCTTTCTTTAAACTGATCTATTTTTTAATTGATTGGTTATGGTGTAAATATTACATAGCTTTAACTGATAATCTGATTACTTTATTTCGTGTGTCTATGATGATGATGATAGTTACAGAACCGACGAACTAGTCTGTACCCGATCAACACCGAaACCCGTGGCGTCTTCGACCTCAATGGCGTCTGGAACTTCAAGCTGGACTACGGGAAAGGACTGGAAGAGAAGTGGTACGAAAGCAAGCTGACCGACACTATTAGTATGGCCGTCCCAAGCAGTTACAATGACATTGGCGTGACCAAGGAAATCCGCAACCATATCGGATATGTCTGGTACGAACGTGAGTTCACGGTGCCGGCCTATCTGAAGGATCAGCGTATCGTGCTCCGCTTCGGCTCTGCAACTCACAAAGCAATTGTCTATGTCAATGGTGAGCTGGTCGTGGAGCACAAGGGCGGATTCCTGCCATTCGAAGCGGAAATCAACAACTCGCTGCGTGATGGCATGAATCGCGTCACCGTCGCCGTGGACAACATCCTCGACGATAGCACCCTCCCGGTGGGGCTGTACAGCGAGCGCCACGAAGAGGGCCTCGGAAAAGTCATTCGTAACAAGCCGAACTTCGACTTCTTCAACTATGCAGGCCTGCACCGTCCGGTGAAAATCTACACGACCCCGTTTACGTACGTCGAGGACATCTCGGTTGTGACCGACTTCAATGGCCCAACCGGGACTGTGACCTATACGGTGGACTTTCAAGGCAAAGCCGAGACtGTGAAAGTGTCGGTCGTGGATGAGGAAGGCAAAGTGGTCGCAAGCACCGAGGGCCTGAGCGGTAACGTGGAGATTCCGAATGTCATCCTCTGGGAACCACTGAACACGTATCTCTACCAGATCAAAGTGGAACTGGTGAACGACGGACTGACCATCGATGTCTATGAAGAGCCGTTCGGCGTGCGGACCGTGGAAGTCAACGACGGCAAGTTCCTCATCAACAACAAACCGTTCTACTTCAAGGGCTTTGGCAAACATGAGGACACTCCTATCAACGGCCGTGGCTTTAACGAAGCGAGCAATGTGATGGATTTCAATATCCTCAAATGGATCGGCGCCAACAGCTTCCGGACCGCACACTATCCGTACTCTGAAGAGTTGATGCGTCTTGCGGATCGCGAGGGTCTGGTCGTGATCGACGAGACTCCGGCAGTTGGCGTGCACCTCAACTTCATGGCCACCACGGGACTCGGCGAAGGCAGCGAGCGCGTCAGTACCTGGGAGAAGATTCGGACGTTTGAGCACCATCAAGACGTTCTCCGTGAACTGGTGTCTCGTGACAAGAACCATCCAAGCGTCGTGATGTGGAGCATCGCCAACGAGGCGGCGACTGAGGAAGAGGGCGCGTACGAGTACTTCAAGCCGTTGGTGGAGCTGACCAAGGAACTCGACCCACAGAAGCGTCCGGTCACGATCGTGCTGTTTGTGATGGCTACCCCGGAGACGGACAAAGTCGCCGAACTGATTGACGTCATCGCGCTCAATCGCTATAACGGATGGTACTTCGATGGCGGTGATCTCGAAGCGGCCAAAGTCCATCTCCGCCAGGAATTTCACGCGTGGAACAAGCGTTGCCCAGGAAAGCCGATCATGATCACTGAGTACGGCGCAGACACCGTTGCGGGCTTTCACGACATTGATCCAGTGATGTTCACCGAGGAATATCAAGTCGAGTACTACCAGGCGAACCACGTCGTGTTCGATGAGTTTGAGAACTTCGTGGGTGAGCAAGCGTGGAACTTCGCGGACTTCGCGACCTCTCAGGGCGTGATGCGCGTCCAAGGAAACAAGAAGGGCGTGTTCACTCGTGACCGCAAGCCGAAGCTCGCCGCGCACGTCTTTCGCGAGCGCTGGACCAACATTCCAGATTTCGGCTACAAGAACGCTAGCCATCACCATCACCATCACGTGTGAATTGGTGACCAGCTCGAATTTCCCCGATCGTTCAAACATTTGGCAATAAAGTTTCTTAAGATTGAATCCTGTTGCCGGTCTTGCGATGATTATCATATAATTTCTGTTGAATTACGTTAAGCATGTAATAATTAACATGTAATGCATGACGTTATTTATGAGATGGGTTTTTATGATTAGAGTCCCGCAATTATACATTTAATACGCGATAGAAAACAAAATATAGCGCGCAAACTAGGATAAATTATCGCGCGCGGTGTCATCTATGTTACTAGATCGGGAATTAAACTATCAGTGTTTGACAGGATATATTGGCGGGTAAACCTAAGAGAAAAGAGCGTTTATTAGAATAACGGATATTTAAAAGGGCGTGAAAAGGTTTATCCGTTCGTCCATTTGTATGTGCATGCCAACCACAGGGTTCCCCTCGGGATCAAAGTACTTTGATCCAACCCCTCCGCTGCTATAGTGCAGTCGGCTTCTGACGTTCAGTGCAGCCGTCTTCTGAAAACGACATGTCGCACAAGTCCTAAGTTACGCGACAGGCTGCCGCCCTGCCCTTTTCCTGGCGTTTTCTTGTCGCGTGTTTTAGTCGCATAAAGTAGAATACTTGCGACTAGAACCGGAGACATTACGCCATGAACAAGAGCGCCGCCGCTGGCCTGCTGGGCTATGCCCGCGTCAGCACCGACGACCAGGACTTGACCAACCAACGGGCCGAACTGCACGCGGCCGGCTGCACCAAGCTGTTTTCCGAGAAGATCACCGGCACCAGGCGCGACCGCCCGGAGCTGGCCAGGATGCTTGACCACCTACGCCCTGGCGACGTTGTGACAGTGACCAGGCTAGACCGCCTGGCCCGCAGCACCCGCGACCTACTGGACATTGCCGAGCGCATCCAGGAGGCCGGCGCGGGCCTGCGTAGCCTGGCAGAGCCGTGGGCCGACACCACCACGCCGGCCGGCCGCATGGTGTTGACCGTGTTCGCCGGCATTGCCGAGTTCGAGCGTTCCCTAATCATCGACCGCACCCGGAGCGGGCGCGAGGCCGCCAAGGCCCGAGGCGTGAAGTTTGGCCCCCGCCCTACCCTCACCCCGGCACAGATCGCGCACGCCCGCGAGCTGATCGACCAGGAAGGCCGCACCGTGAAAGAGGCGGCTGCACTGCTTGGCGTGCATCGCTCGACCCTGTACCGCGCACTTGAGCGCAGCGAGGAAGTGACGCCCACCGAGGCCAGGCGGCGCGGTGCCTTCCGTGAGGACGCATTGACCGAGGCCGACGCCCTGGCGGCCGCCGAGAATGAACGCCAAGAGGAACAAGCATGAAACCGCACCAGGACGGCCAGGACGAACCGTTTTTCATTACCGAAGAGATCGAGGCGGAGATGATCGCGGCCGGGTACGTGTTCGAGCCGCCCGCGCACGTCTCAACCGTGCGGCTGCATGAAATCCTGGCCGGTTTGTCTGATGCCAAGCTGGCGGCCTGGCCGGCCAGCTTGGCCGCTGAAGAAACCGAGCGCCGCCGTCTAAAAAGGTGATGTGTATTTGAGTAAAACAGCTTGCGTCATGCGGTCGCTGCGTATATGATGCGATGAGTAAATAAACAAATACGCAAGGGGAACGCATGAAGGTTATCGCTGTACTTAACCAGAAAGGCGGGTCAGGCAAGACGACCATCGCAACCCATCTAGCCCGCGCCCTGCAACTCGCCGGGGCCGATGTTCTGTTAGTCGATTCCGATCCCCAGGGCAGTGCCCGCGATTGGGCGGCCGTGCGGGAAGATCAACCGCTAACCGTTGTCGGCATCGACCGCCCGACGATTGACCGCGACGTGAAGGCCATCGGCCGGCGCGACTTCGTAGTGATCGACGGAGCGCCCCAGGCGGCGGACTTGGCTGTGTCCGCGATCAAGGCAGCCGACTTCGTGCTGATTCCGGTGCAGCCAAGCCCTTACGACATATGGGCCACCGCCGACCTGGTGGAGCTGGTTAAGCAGCGCATTGAGGTCACGGATGGAAGGCTACAAGCGGCCTTTGTCGTGTCGCGGGCGATCAAAGGCACGCGCATCGGCGGTGAGGTTGCCGAGGCGCTGGCCGGGTACGAGCTGCCCATTCTTGAGTCCCGTATCACGCAGCGCGTGAGCTACCCAGGCACTGCCGCCGCCGGCACAACCGTTCTTGAATCAGAACCCGAGGGCGACGCTGCCCGCGAGGTCCAGGCGCTGGCCGCTGAAATTAAATCAAAACTCATTTGAGTTAATGAGGTAAAGAGAAAATGAGCAAAAGCACAAACACGCTAAGTGCCGGCCGTCCGAGCGCACGCAGCAGCAAGGCTGCAACGTTGGCCAGCCTGGCAGACACGCCAGCCATGAAGCGGGTCAACTTTCAGTTGCCGGCGGAGGATCACACCAAGCTGAAGATGTACGCGGTACGCCAAGGCAAGACCATTACCGAGCTGCTATCTGAATACATCGCGCAGCTACCAGAGTAAATGAGCAAATGAATAAATGAGTAGATGAATTTTAGCGGCTAAAGGAGGCGGCATGGAAAATCAAGAACAACCAGGCACCGACGCCGTGGAATGCCCCATGTGTGGAGGAACGGGCGGTTGGCCAGGCGTAAGCGGCTGGGTTGTCTGCCGGCCCTGCAATGGCACTGGAACCCCCAAGCCCGAGGAATCGGCGTGACGGTCGCAAACCATCCGGCCCGGTACAAATCGGCGCGGCGCTGGGTGATGACCTGGTGGAGAAGTTGAAGGCCGCGCAGGCCGCCCAGCGGCAACGCATCGAGGCAGAAGCACGCCCCGGTGAATCGTGGCAAGCGGCCGCTGATCGAATCCGCAAAGAATCCCGGCAACCGCCGGCAGCCGGTGCGCCGTCGATTAGGAAGCCGCCCAAGGGCGACGAGCAACCAGATTTTTTCGTTCCGATGCTCTATGACGTGGGCACCCGCGATAGTCGCAGCATCATGGACGTGGCCGTTTTCCGTCTGTCGAAGCGTGACCGACGAGCTGGCGAGGTGATCCGCTACGAGCTTCCAGACGGGCACGTAGAGGTTTCCGCAGGGCCGGCCGGCATGGCCAGTGTGTGGGATTACGACCTGGTACTGATGGCGGTTTCCCATCTAACCGAATCCATGAACCGATACCGGGAAGGGAAGGGAGACAAGCCCGGCCGCGTGTTCCGTCCACACGTTGCGGACGTACTCAAGTTCTGCCGGCGAGCCGATGGCGGAAAGCAGAAAGACGACCTGGTAGAAACCTGCATTCGGTTAAACACCACGCACGTTGCCATGCAGCGTACGAAGAAGGCCAAGAACGGCCGCCTGGTGACGGTATCCGAGGGTGAAGCCTTGATTAGCCGCTACAAGATCGTAAAGAGCGAAACCGGGCGGCCGGAGTACATCGAGATCGAGCTAGCTGATTGGATGTACCGCGAGATCACAGAAGGCAAGAACCCGGACGTGCTGACGGTTCACCCCGATTACTTTTTGATCGATCCCGGCATCGGCCGTTTTCTCTACCGCCTGGCACGCCGCGCCGCAGGCAAGGCAGAAGCCAGATGGTTGTTCAAGACGATCTACGAACGCAGTGGCAGCGCCGGAGAGTTCAAGAAGTTCTGTTTCACCGTGCGCAAGCTGATCGGGTCAAATGACCTGCCGGAGTACGATTTGAAGGAGGAGGCGGGGCAGGCTGGCCCGATCCTAGTCATGCGCTACCGCAACCTGATCGAGGGCGAAGCATCCGCCGGTTCCTAATGTACGGAGCAGATGCTAGGGCAAATTGCCCTAGCAGGGGAAAAAGGTCGAAAAGGTCTCTTTCCTGTGGATAGCACGTACATTGGGAACCCAAAGCCGTACATTGGGAACCGGAACCCGTACATTGGGAACCCAAAGCCGTACATTGGGAACCGGTCACACATGTAAGTGACTGATATAAAAGAGAAAAAAGGCGATTTTTCCGCCTAAAACTCTTTAAAACTTATTAAAACTCTTAAAACCCGCCTGGCCTGTGCATAACTGTCTGGCCAGCGCACAGCCGAAGAGCTGCAAAAAGCGCCTACCCTTCGGTCGCTGCGCTCCCTACGCCCCGCCGCTTCGCGTCGGCCTATCGCGGCCGCTGGCCGCTCAAAAATGGCTGGCCTACGGCCAGGCAATCTACCAGGGCGCGGACAAGCCGCGCCGTCGCCACTCGACCGCCGGCGCCCACATCAAGGCACCCTGCCTCGCGCGTTTCGGTGATGACGGTGAAAACCTCTGACACATGCAGCTCCCGGAGACGGTCACAGCTTGTCTGTAAGCGGATGCCGGGAGCAGACAAGCCCGTCAGGGCGCGTCAGCGGGTGTTGGCGGGTGTCGGGGCGCAGCCATGACCCAGTCACGTAGCGATAGCGGAGTGTATACTGGCTTAACTATGCGGCATCAGAGCAGATTGTACTGAGAGTGCACCATATGCGGTGTGAAATACCGCACAGATGCGTAAGGAGAAAATACCGCATCAGGCGCTCTTCCGCTTCCTCGCTCACTGACTCGCTGCGCTCGGTCGTTCGGCTGCGGCGAGCGGTATCAGCTCACTCAAAGGCGGTAATACGGTTATCCACAGAATCAGGGGATAACGCAGGAAAGAACATGTGAGCAAAAGGCCAGCAAAAGGCCAGGAACCGTAAAAAGGCCGCGTTGCTGGCGTTTTTCCATAGGCTCCGCCCCCCTGACGAGCATCACAAAAATCGACGCTCAAGTCAGAGGTGGCGAAACCCGACAGGACTATAAAGATACCAGGCGTTTCCCCCTGGAAGCTCCCTCGTGCGCTCTCCTGTTCCGACCCTGCCGCTTACCGGATACCTGTCCGCCTTTCTCCCTTCGGGAAGCGTGGCGCTTTCTCATAGCTCACGCTGTAGGTATCTCAGTTCGGTGTAGGTCGTTCGCTCCAAGCTGGGCTGTGTGCACGAACCCCCCGTTCAGCCCGACCGCTGCGCCTTATCCGGTAACTATCGTCTTGAGTCCAACCCGGTAAGACACGACTTATCGCCACTGGCAGCAGCCACTGGTAACAGGATTAGCAGAGCGAGGTATGTAGGCGGTGCTACAGAGTTCTTGAAGTGGTGGCCTAACTACGGCTACACTAGAAGGACAGTATTTGGTATCTGCGCTCTGCTGAAGCCAGTTACCTTCGGAAAAAGAGTTGGTAGCTCTTGATCCGGCAAACAAACCACCGCTGGTAGCGGTGGTTTTTTTGTTTGCAAGCAGCAGATTACGCGCAGAAAAAAAGGATCTCAAGAAGATCCTTTGATCTTTTCTACGGGGTCTGACGCTCAGTGGAACGAAAACTCACGTTAAGGGATTTTGGTCATGCATTCTAGGTACTAAAACAATTCATCCAGTAAAATATAATATTTTATTTTCTCCCAATCAGGCTTGATCCCCAGTAAGTCAAAAAATAGCTCGACATACTGTTCTTCCCCGATATCCTCCCTGATCGACCGGACGCAGAAGGCAATGTCATACCACTTGTCCGCCCTGCCGCTTCTCCCAAGATCAATAAAGCCACTTACTTTGCCATCTTTCACAAAGATGTTGCTGTCTCCCAGGTCGCCGTGGGAAAAGACAAGTTCCTCTTCGGGCTTTTCCGTCTTTAAAAAATCATACAGCTCGCGCGGATCTTTAAATGGAGTGTCTTCTTCCCAGTTTTCGCAATCCACATCGGCCAGATCGTTATTCAGTAAGTAATCCAATTCGGCTAAGCGGCTGTCTAAGCTATTCGTATAGGGACAATCCGATATGTCGATGGAGTGAAAGAGCCTGATGCACTCCGCATACAGCTCGATAATCTTTTCAGGGCTTTGTTCATCTTCATACTCTTCCGAGCAAAGGACGCCATCGGCCTCACTCATGAGCAGATTGCTCCAGCCATCATGCCGTTCAAAGTGCAGGACCTTTGGAACAGGCAGCTTTCCTTCCAGCCATAGCATCATGTCCTTTTCCCGTTCCACATCATAGGTGGTCCCTTTATACCGGCTGTCCGTCATTTTTAAATATAGGTTTTCATTTTCTCCCACCAGCTTATATACCTTAGCAGGAGACATTCCTTCCGTATCTTTTACGCAGCGGTATTTTTCGATCAGTTTTTTCAATTCCGGTGATATTCTCATTTTAGCCATTTATTATTTCCTTCCTCTTTTCTACAGTATTTAAAGATACCCCAAGAAGCTAATTATAACAAGACGAACTCCAATTCACTGTTCCTTGCATTCTAAAACCTTAAATACCAGAAAACAGCTTTTTCAAAGTTGTTTTCAAAGTTGGCGTATAACATAGTATCGACGGAGCCGATTTTGAAACCGCGGTGATCACAGGCAGCAACGCTCTGTCATCGTTACAATCAACATGCTACCCTCCGCGAGATCATCCGTGTTTCAAACCCGGCAGCTTAGTTGCCGTTCTTCCGAATAGCATCGGTAACATGAGCAAAGTCTGCCGCCTTACAACGGCTCTCCCGCTGACGCCGTCCCGGACTGATGGGCTGCCTGTATCGAGTGGTGATTTTGTGCCGAGCTGCCGGTCGGGGAGCTGTTGGCTGGCTGGTGGCAGGATATATTGTGGTGTAAACAAATTGACGCTTAGACAACTTAATAACACATTGCGGACGTTTTTAATGTACTGAATTAACGCCGAATTAATTCGGGGGATCTGGATTTTAGTACTGGATTTTGGTTTTAGGAATTAGAAATTTTATTGATAGAAGTATTTTACAAATACAAATACATACTAAGGGTTTCTTATATGCTCAACACATGAGCGAAACCCTATAGGAACCCTAATTCCCTTATCTGGGAACTACTCACACATTATTATGGAGAAACTCGAGCTTGTCGATCGACAGATCCGGTCGGCATCTACTCTATTTCTTTGCCCTCGGACGAGTGCTGGGGCGTCGGTTTCCACTATCGGCGAGTACTTCTACACAGCCATCGGTCCAGACGGCCGCGCTTCTGCGGGCGATTTGTGTACGCCCGACAGTCCCGGCTCCGGATCGGACGATTGCGTCGCATCGACCCTGCGCCCAAGCTGCATCATCGAAATTGCCGTCAACCAAGCTCTGATAGAGTTGGTCAAGACCAATGCGGAGCATATACGCCCGGAGTCGTGGCGATCCTGCAAGCTCCGGATGCCTCCGCTCGAAGTAGCGCGTCTGCTGCTCCATACAAGCCAACCACGGCCTCCAGAAGAAGATGTTGGCGACCTCGTATTGGGAATCCCCGAACATCGCCTCGCTCCAGTCAATGACCGCTGTTATGCGGCCATTGTCCGTCAGGACATTGTTGGAGCCGAAATCCGCGTGCACGAGGTGCCGGACTTCGGGGCAGTCCTCGGCCCAAAGCATCAGCTCATCGAGAGCCTGCGCGACGGACGCACTGACGGTGTCGTCCATCACAGTTTGCCAGTGATACACATGGGGATCAGCAATCGCGCATATGAAATCACGCCATGTAGTGTATTGACCGATTCCTTGCGGTCCGAATGGGCCGAACCCGCTCGTCTGGCTAAGATCGGCCGCAGCGATCGCATCCATAGCCTCCGCGACCGGTTGTAGAACAGCGGGCAGTTCGGTTTCAGGCAGGTCTTGCAACGTGACACCCTGTGCACGGCGGGAGATGCAATAGGTCAGGCTCTCGCTAAACTCCCCAATGTCAAGCACTTCCGGAATCGGGAGCGCGGCCGATGCAAAGTGCCGATAAACATAACGATCTTTGTAGAAACCATCGGCGCAGCTATTTACCCGCAGGACATATCCACGCCCTCCTACATCGAAGCTGAAAGCACGAGATTCTTCGCCCTCCGAGAGCTGCATCAGGTCGGAGACGCTGTCGAACTTTTCGATCAGAAACTTCTCGACAGACGTCGCGGTGAGTTCAGGCTTTTTCATATCTCATTGCCCCCCGGGATCTGCGAAAGCTCGAGAGAGATAGATTTGTAGAGAGAGACTGGTGATTTCAGCGTGTCCTCTCCAAATGAAATGAACTTCCTTATATAGAGGAAGGTCTTGCGAAGGATAGTGGGATTGTGCGTCATCCCTTACGTCAGTGGAGATATCACATCAATCCACTTGCTTTGAAGACGTGGTTGGAACGTCTTCTTTTTCCACGATGCTCCTCGTGGGTGGGGGTCCATCTTTGGGACCACTGTCGGCAGAGGCATCTTGAACGATAGCCTTTCCTTTATCGCAATGATGGCATTTGTAGGTGCCACCTTCCTTTTCTACTGTCCTTTTGATGAAGTGACAGATAGCTGGGCAATGGAATCCGAGGAGGTTTCCCGATATTACCCTTTGTTGAAAAGTCTCAATAGCCCTTTGGTCTTCTGAGACTGTATCTTTGATATTCTTGGAGTAGACGAGAGTGTCGTGCTCCACCATGTTATCACATCAATCCACTTGCTTTGAAGACGTGGTTGGAACGTCTTCTTTTTCCACGATGCTCCTCGTGGGTGGGGGTCCATCTTTGGGACCACTGTCGGCAGAGGCATCTTGAACGATAGCCTTTCCTTTATCGCAATGATGGCATTTGTAGGTGCCACCTTCCTTTTCTACTGTCCTTTTGATGAAGTGACAGATAGCTGGGCAATGGAATCCGAGGAGGTTTCCCGATATTACCCTTTGTTGAAAAGTCTCAATAGCCCTTTGGTCTTCTGAGACTGTATCTTTGATATTCTTGGAGTAGACGAGAGTGTCGTGCTCCACCATGTTGGCAAGCTGCTCTAGCCAATACGCAAACCGCCTCTCCCCGCGCGTTGGCCGATTCATTAATGCAGCTGGCACGACAGGTTTCCCGACTGGAAAGCGGGCAGTGAGCGCAACGCAATTAATGTGAGTTAGCTCACTCATTAGGCACCCCAGGCTTTACACTTTATGCTTCCGGCTCGTATGTTGTGTGGAATTGTGAGCGGATAACAATTTCACACAGGAAACAGCTATGACCATGATTACGAATTCGAGCTC
