# Supplementary material for: One-step generation of composite soybean plants with transgenic roots by Agrobacterium rhizogenes-mediated transformation
Source: BMC Plant Biol. 2020 May 12;20:208. doi: 10.1186/s12870-020-02421-4 (PMC7333419; doi:10.1186/s12870-020-02421-4)
Supplement: Supplementary file 9 — Additional file 9: Table S1. A comparison of hairy roots produced by one-step and two-step method. [file 12870_2020_2421_MOESM9_ESM.docx]

Table S1 A comparison of hairy roots produced by one-step and two-step method.

| Transformation method | Total length of hairy roots  (per seedling, mm) | Total weight of hairy roots  (per seedling, mg) |
| --- | --- | --- |
| one-step (Cutting) | 242.73±87.59^A^ | 470±142^A^ |
| two-step (Stabbing) | 5.31±2.86^B^ | 6.53±3.78^B^ |

The data is at 16 dpi. Letter A and B represented by different letters are very significantly different at the p=0.01 value given. Values are mean ± SD for three independent replicates (n=15).
