# Supplementary material for: One-step generation of composite soybean plants with transgenic roots by Agrobacterium rhizogenes-mediated transformation
Source: BMC Plant Biol. 2020 May 12;20:208. doi: 10.1186/s12870-020-02421-4 (PMC7333419; doi:10.1186/s12870-020-02421-4)
Supplement: Supplementary file 10 — Additional file 10: Table S2. All primers sequences used in this paper. [file 12870_2020_2421_MOESM10_ESM.docx]

Table S2 All primers sequences used in this paper.

| primers name | Sequence (5’-3’) | purposes |
| --- | --- | --- |
| 13051F | ACAGGAATTCGAACTCGCCGTAAAGACTGG | *GUS* replaced of *HptII in* pGSE401 |
| 13051R | TGGTCTCTTTCGGTGTTGATCGGGTACAG | *GUS* replaced of *HptII in* pGSE401 |
| 13052F | AGGTCTCACGAAACCCGTGGCGTCTTCGACC | *GUS* replaced of *HptII in* pGSE401 |
| 13052R | AGGTCTCCAGTCTCGGCTTTGCCTTGA | *GUS* replaced of *HptII in* pGSE401 |
| 13053F | AGGTCTCAGACTGTGAAAGTGTCGGTCGTGGA | *GUS* replaced of *HptII in* pGSE401 |
| 13053R | CTGTGCGATCGCGATAGTTTAATTCCCGATC | *GUS* replaced of *HptII in* pGSE401 |
| 13051F | ACAGGAATTCGAACTCGCCGTAAAGACTGG | *GUS* replaced of *HptII in* pGSE401 |
| KtRfg1F | ATTGACAGTAAGCCTTACTACCT | Construction of CRISPR/Cas9-mediated *Rfg1* gene knockout |
| KtRfg1R | AAACAGGTAGTAAGGCTTACTGT | Construction of CRISPR/Cas9-mediated *Rfg1* gene knockout |
| YAO18F | CTGCAGGTACCTCTGAATCGAGCTTTCGGAA | PCR amplify YAO promoter, with *Kpn*I digestion site was underlined |
| YAO19R | CTGAACCATGGTCTCTCTCACTCCCTCTTAG | PCR amplify YAO promoter, with *Nco*I digestion site was underlined |
| ktR14F | TTGACGGGTGCAAATGTTTA | Sequencing primer for identification on CRISPR/Cas9-mediated gene disruption |
| ktR14R | TTGCTGATCGAACCACTCTG | Sequencing primer for identification on CRISPR/Cas9-mediated gene disruption |
| GUSF | TCTCCGTGAACTGGTGTCTC | PCR primer for amplification *GUS* gene |
| GUSR | TTCGCCTGGTAGTACTCGAC | PCR primer for amplification *GUS* gene |
| GmActinF | GAGCTATGAATTGCCTGATGG | RT-PCR primer for amplification *actin* gene in soybean |
| GmActinR | CGTTTCATGAATTCCAGTAGC | RT-PCR primer for amplification *actin* gene in soybean |
| RTGusF | TGACCATGGTAGATCTGAGG | RT-PCR primer for amplification *GUS* gene in soybean |
| RTGusR | TTTGCCTTGAAAGTCCACCG | RT-PCR primer for amplification *GUS* gene in soybean |
| PTA | CCGGATCCTCTAGAGCGGCCGC(T)17 | RT-PCR primer for first strand cDNA synthesis |
| SPNos1 | CACGTGTGAATTGGTGACCA (333bp from RB) | TAIL-PCR Specific primer for Nos terminator |
| SPNos2 | GAATCCTGTTGCCGGTCTTG (261bp from RB) | TAIL-PCR SpesTAIL-PCR Specific primer for Nos terminator |
| SPNos3 | GATTAGAGTCCCGCAATTATAC (150bp from RB) | TAIL-PCR Specific primer for Nos terminator |
| LAD1-1 | ACGATGGACTCCAGAGCGGCCGC(G/C/A)N(G/C/A)NNNGGAA | TAIL-PCR Longer AD (LAD) primer |
| LAD1-2 | ACGATGGACTCCAGAGCGGCCGC(G/C/T)N(G/C/T)NNNGGTT | TAIL-PCR Longer AD (LAD) primer |
| LAD1-3 | ACGATGGACTCCAGAGCGGCCGC(G/C/A)N(G/C/A)NNNCCAC | TAIL-PCR Longer AD (LAD) primer |
| LAD1-4 | ACGATGGACTCCAGAGCGGCCGC(G/C/A)(G/C/A)N(G/C/A)NNNCCAA | TAIL-PCR Longer AD (LAD) primer |
| AC | ACGATGGACTCCAGAG | TAIL-PCR primer specific to LAD primers |
